# Multi-Targeting Valproic Acid Conjugates as Potent Agents Against Inflammation and Hyperlipidemia

**DOI:** 10.3390/molecules30112339

**Published:** 2025-05-27

**Authors:** Panagiotis Theodosis-Nobelos, Eleni A. Rekka

**Affiliations:** 1 Department of Pharmacy, School of Health Sciences, Frederick University, 1036 Nicosia, Cyprus; 2 Department of Pharmaceutical Chemistry, School of Pharmacy, Aristotelian University of Thessaloniki, 54124 Thessaloniki, Greece; rekka@pharm.auth.gr

**Keywords:** anti-inflammatory agents, valproic acid derivatives, inflammation, NSAIDs, lipoxygenase inhibition, antioxidant, hyperlipidemia, triglycerides decrease, cholesterol reduction

## Abstract

Novel derivatives of valproic acid with biologically active moieties, such as thiomorpholine, 4-aminopyridine, serine methyl ester, trolox and the cinnamic acid derivative [(*E*)-3-(3,5-di-*tert*-butyl-4-hydroxyphenyl)acrylic acid], were synthesized at satisfactory yields. The conjugation of these moieties was based on the rationale of design and evaluation of compounds with selected structural characteristics, aiming at derivatives with multiple targets. These compounds reduced acute inflammation considerably and, in most cases, more than several highly used, well-known, non-steroidal anti-inflammatory drugs. They also offered the inhibition of soybean lipoxygenase, and some of them (compounds **5** and **6**) possessed radical scavenging and lipid peroxidation attenuating effects. Their antioxidant capacity was several times higher than that of the established antioxidant trolox. All the tested compounds decreased plasma lipid markers in tyloxapol-induced hyperlipidemia in rats. Compound **2** resulted in 71.1%, 52.8% and 79.1% decrease in total cholesterol, triglycerides and LDL-cholesterol, respectively, at 150 μmol/kg (*i.p*.). The effect on total and LDL cholesterol is comparable or equal to that of simvastatin, a hypocholesterolemic 3-hydroxy-3-methylglutaryl coenzyme A reductase (HMG-CoA) inhibitor, however, with additionally great triglyceride-decreasing effect compared to simvastatin. Thus, the synthesized compounds may be a valuable addition to multi-functional agents acting against various degenerative disorders that implicate inflammation and lipid derangement.

## 1. Introduction

Although it is a physiological defensive reaction of the organism, inflammation is highly associated with acute and chronic conditions, such as cardiovascular diseases (CVDs), hypertension, diabetes, cancer and aging [1,2]. A critical step in this process is the inflammatory neutrophil, monocyte and leucocyte infiltration in the suffering area, with the participation of various chemokines, adhesion molecules and chemical mediators such as eicosanoids, nitric oxide and cytokines [3]. The activated phagocytic cells and pro-inflammatory cytokines increase the synthesis of reactive oxygen, nitrogen and chlorine species (ROS, RNS, RClS), with further initiation of oxidative modifications and tissue injury locally [4]. During oxidative stress (OS), these reactive species induce the activation of transcription factors, like NF-κB (nuclear factor-κB), and inflammasome triggers innate immune defense. This series of events leads to IL-1 and 6 secretion and inflammatory propagation [5], indicating the interrelation of oxidative and inflammatory processes. Hypercholesterolemia is closely related to CVDs, the development of atherosclerosis and other degenerative disorders, via the induction of inflammatory mechanisms, oxidative and nitrosative stress [6].

Valproic acid (VA) is a drug with antiepileptic, anti-migraine and mood stabilizing properties. It expresses its GABAergic and anti-excitatory activity via ion channel modulation on glutamatergic neurons [7,8]. VA seems also to lead to the inhibition of histone deacetylase activity and to possess anti-inflammatory, cell proliferative, anti-apoptotic and antioxidant properties [9,10]. It also inhibits the production of TNF-α, IL-6, IL-8 and other cytokines induced by lipopolysaccharides [11], whilst various mechanisms, including histone deacetylase activity and leucocyte migration inhibition, are important for its anti-inflammatory effect [12,13]. In addition, VA can attenuate the damage in multiple organs via the implication of various mechanisms and signaling pathways [14,15]. VA reduces ischemia–reperfusion injury in the brain and kidneys, with reduction in myeloperoxidase, 4-hydroxy-2-nonenal and 8-hydroxydeoxyguanosine levels and decreases the activation of various inflammation-related factors such as the translocation of NF-κB and matrix metalloproteinase 9 production in vivo [16,17]. These findings indicate the potentially pluripotent effects of VA in various conditions through oxidative stress and neutrophil infiltration decrement. The neuroprotective effect is the most prominent of VA activities [18]. The central nervous system is relatively prone to OS, due to an insufficient supply of endogenous antioxidant defense systems, leading to mitochondrial dysfunction, protein aggregation and activation of microglia and astrocytes, which contribute to neuroinflammation in neurodegenerative disorders, such as AD [19,20]. VA may contribute towards the protection against these conditions, although toxicity issues may arise during its application, with mainly hepatotoxic, teratogenic and energy-deranging effects [21]. However, amidated derivatives of VA seem to be less toxic, affecting key enzymes to a lesser extent, offering cell preservation in the liver and the embryo [21,22]. This finding, combined with the narrow array of experimental evidence of VA, until nowadays, mainly concerning the convulsion and sedation level, leaves the area of search and application of VA at inflammation, lipid levels’ improvement and OS unexplored [22].

Lipoxygenases (LOXs) can exert inflammatory actions and induce the progression of atherosclerotic plaques and lipid tissue inflammation, whilst their inhibition can partly limit the undesired gastric and renal effects of NSAIDs [cycloxygenase 1 and 2 (COX-1 and 2) inhibitors, non-steroidal anti-inflammatory drugs] [23,24,25]. Cinnamic acid and cinnamaldehyde also seem to offer a decrease in the inflammatory factors of COX-2, NF-κB and iNOS (inducible nitric oxide synthase) cellular production [26]. Some of their analogues, especially hydroxylated cinnamic acids, possess increased anti-inflammatory and hypolipidemic activity [27]. Furthermore, thiomorpholine derivatives exert various activities implicating metabolic conditions and the cardiovascular system, including hypolipidemic and antioxidant effects [28,29], while 4-aminopyridine, a potassium channel modulator, possesses neuroprotective effects, thus resembling tacrine derivatives [30]. Additionally, as far as the amino acids are concerned, the serine offered increased survival rate and reduction in cytokine production, decreasing macrophage stimulation and inducing antioxidant factors such as heme oxygenase-1 and the nuclear-related factor 2 in endothelial cells [31,32].

**Scheme 1:**
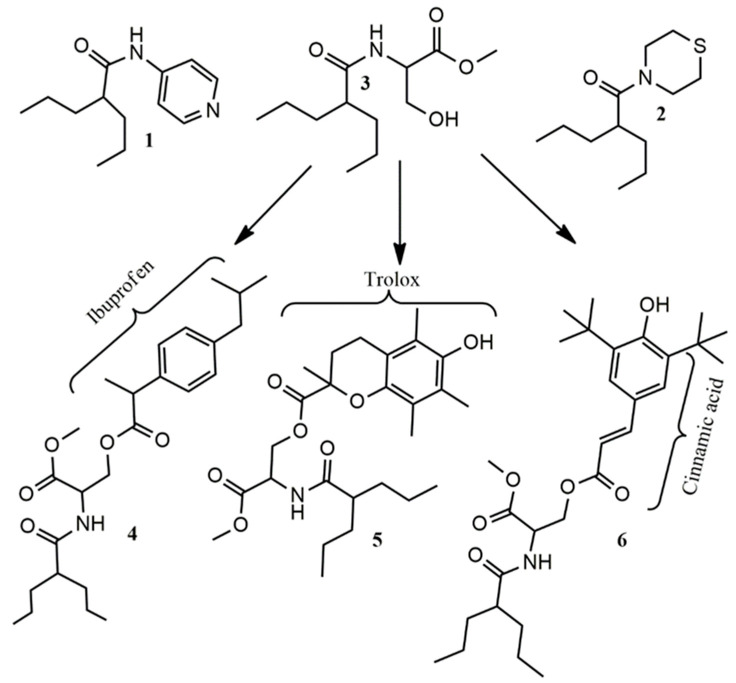
The synthesized compounds **1**–**6**.

**Scheme 2:**
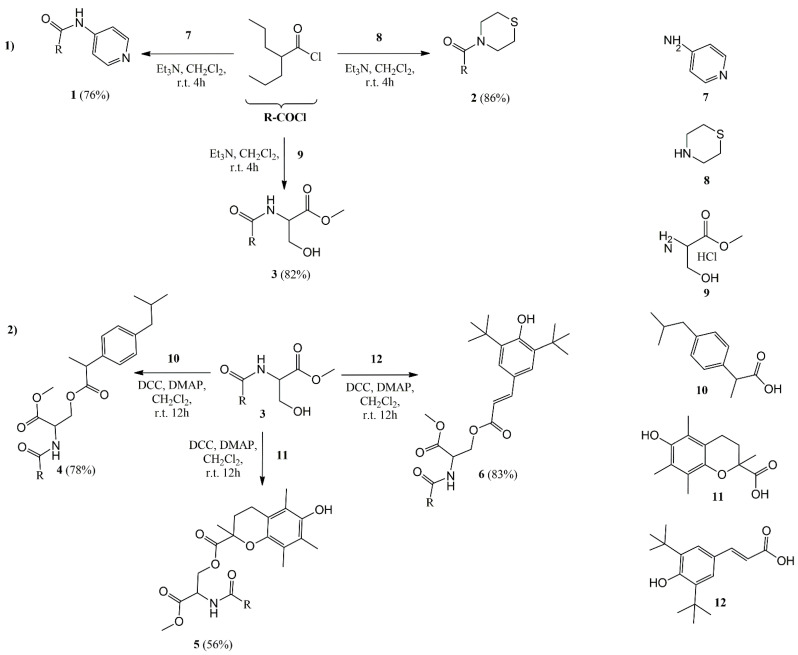
Synthetic procedures for compounds **1**–**6**: Method (1): A solution of amines **7** and **8** or a suspension of compound **9** was mixed with 2-propylpentanoyl chloride (R-COCl) and DMAP, at 0–5 °C for 15 min, and afterwards for 4 h at room temperature, yielding compounds **1**–**3**; Method (2): To a solution of compound **3**, solutions of the corresponding acids (compounds **10**–**12**), DMAP and DCC were mixed for 12 h at room temperature yielding compounds **4**–**6** (DCC: N,N′-dicyclohexylcarbodiimide; DMAP: N,N-dimethylaminopyridine; r.t.: room temperature).

In view of the above-mentioned evidence, valproic acid conjugated with cytoprotective, antioxidant, anti-inflammatory and hypolipidemic agents could be successful in the treatment of diseases that include multiple mechanisms of induction, as the neurodegenerative disorders. Therefore, we synthesized and tested a series of amides of valproic acid with 4-aminopyridine, thiomorpholine and serine methyl ester, compounds **1**, **2** and **3** (Scheme 1). In addition, esterification of compound **3** with ibuprofen (**10**), trolox (**11**) and (*E*)-3-(3,5-di-*tert*-butyl-4-hydroxyphenyl)acrylic acid (**12**) (Scheme 2) provided derivatives **4**, **5** and **6**.

The anti-inflammatory activity of the compounds, expressed as reduction in rat paw edema produced by carrageenan and effect on the activity of soybean lipoxygenase, was studied. Furthermore, their antioxidant potential was examined via the study of lipid peroxidation inhibition and the stable DPPH radical scavenging. Their hypolipidemic activity in vivo was also tested, since lipid level deterioration is a crucial element for CVD and neurodegeneration [33,34].

## 2. Results and Discussion

### 2.1. Synthesis

Compounds **1**–**3** were synthesized by direct amidation of valproyl chloride (2-propylpentanoyl chloride) with the respective amine **7** or **8** or amine hydrochloride **9**, at room temperature, with excellent yields varying between 65% and 86% (Μethod 1, Scheme 2). Compound **6** was esterified with ibuprofen **10**, trolox **11** and (*E*)-4-hydroxyphenyl)acrylic acid **12**, using DCC at room temperature, to afford the corresponding derivatives **4**–**6**, in 78%, 56% and 83% yields, respectively.

### 2.2. In Vivo Evaluation

#### 2.2.1. Effect on the Inflammatory Edema Production

The in vivo model of carrageenan-produced edema is widely used for the evaluation of the anti-inflammatory activity of compounds [35,36,37]. This edema evolves in three phases. Mast cells’ release of modulators of histamine and serotonin during the first hour is followed by cytokine release (the second hour) and a later phase (2.5–6 h) of increased prostaglandin and leukotriene production. Cellular and vascular responses take place, and the most pronounced rate of increase is recorded after 2 to 5 h [38,39,40]. The edema inhibition (after 3.5 h) by the compounds and the well-known reference NSAID ibuprofen is presented in Table 1.

All compounds offered edema reduction from 29% to 48%. They were relatively more active than ibuprofen, except for compounds **3** and **6**, which were around 20% less active. Although the variation in activity is not wide, an increase in activity is observed when ibuprofen or trolox is incorporated (compounds **4** and **5**, respectively), verifying the potential implication of the antioxidant factor against acute inflammation, whilst compound **3,** which does not contain these moieties, expressed significantly lower activity.

Neutrophil activation and high levels of ROS, RNS and PGE2 (prostaglandin E2) are observed in paw exudate. In the same direction, expression of the inducible NO synthetase (iNOS) leads to an increase in their levels at the final stages of inflammation, underlining the complex nature of the inflammatory progression and the implication of peroxynitrite in oxidative processes [41,42].

We have shown that 4-aminopyridine amides of the widely administered NSAIDS tolfenamic acid and indomethacin can decrease edema considerably, in comparison to the parent acids [43]. Compound **1** had the best result in this experiment. Concerning VA, it has been shown that in approximate doses of 0.6, 1.2 and 2.4 mmol/kg i.p., VA succeeded 15%, 36% and 48% inhibition of carrageenan paw edema within 2 h, whilst, at 2.4 mmol/kg, the effect of VA was comparable to that of 0.28 mmol/kg indomethacin [44]. In our experimental conditions, indomethacin, at 0.15 mmol/kg, demonstrated a 42% reduction [43], comparable to that of the valproic acid derivatives under examination, at the same dose.

#### 2.2.2. Effect on Hyperlipidemia

Hyperlipidemia is a serious risk factor for cardiovascular disease, coronary heart disease and stroke. A reduction in low-density lipoprotein cholesterol (LDL-C) levels reduces the risk of these conditions. Lipid derangement also affects the myocardial function at metabolic, oxidative stress and inflammatory levels, showing the interrelation of the mechanisms of OS, inflammation and metabolic disturbances [45,46,47]. Additionally, increased lipid levels, and especially triglycerides, may lead to peripheral autonomic and CNS neuropathy. Lipid-modifying agents and natural compounds targeting both at OS and inflammation prove beneficial in halting the progression of neuropathy, decreasing, apart from lipid markers [total cholesterol (TC), triglycerides (TG) and LDL-C], other factors, like IL-1 (interleukin-1), TNF-α (tumor necrosis factor α) and oxidized LDL, centrally and at the periphery [48,49,50].

Hyperlipidemia produced via Triton WR 1339 (tyloxapol) administration systemically (200 mg/kg, *i.p*.) results in an increase in lipids (TC, TG and LDL-C) in two phases. The increase in lipids during the first 24 h is linear and sharp, with stabilization and decrease being recorded after this period [51,52]. Compounds acting on the first phase have been shown to interfere with cholesterol synthesis at the cellular level [53,54]. The results of the anti-hyperlipidemic activity of the compounds and the reference simvastatin (hypolipidemic HMG-CoA inhibitor) are given in Table 2.

A statistically significant decrease in all lipidemic indices, for all the compounds, was recorded. Simvastatin profoundly lowered cholesterol, having no effect on plasma triglyceride levels [55,56]. We have reported that thiomorpholine [28,56] derivatives possess hypolipidemic activity, and this is confirmed for compound **2**. Compound **2** seems to offer a similar decrease in LDL and total cholesterol to that of simvastatin and the most profound decrease in triglyceride levels of all compounds. Compound **4** has shown increased activity on the cholesterol content and similar activity on triglycerides with ibuprofen (decrease of 41% for TC, 38% for TG and 41.6% for LDL-C at 300 μmol/kg *i.p*.), although the latter was used at a two times higher dose. Almost 1.5 times higher activity was recorded for compound **4** than ibuprofen, although compound **3** showed almost half of the activity of compound **4** for the LDL-C. Although trolox has been reported to have minor hypolipidemic activity [57], compound **5** exhibited a high decrease in lipidemic markers, especially triglycerides. This may in part be due to the antioxidant activity of the compound, and the lower antioxidant potency may be the reason for the relatively decreased activity of compound **6**, compared to compound **5**, in this experiment. The results of the serine derivatives are in accordance with previous findings [58], where dual antioxidant and anti-inflammatory serine-containing compounds showed increased, comparable to simvastatin, activity in these three lipidemic indices. Serine is crucial for mitochondrial function, affecting acylcarnitine levels, whilst its deprivation is related to increased mitochondrial fragmentation [59,60].

### 2.3. In Vitro Evaluation

#### 2.3.1. Effect on Lipoxygenase

Lipoxygenases (LOXs) are responsible for the dioxygenation of arachidonic acid. They are related to inflammatory processes of the nervous system and the periphery, including adipose tissue [61,62]. Furthermore, lipoxygenase inhibition results in decreased amyloid plaque formation and Aβ levels and improved tau protein metabolism [63]. Soybean lipoxygenase is a widely applied, in the literature, model for the anti-inflammatory activity evaluation of compounds in vitro [64,65,66]. The inhibitory activity of the compounds on soybean LOX is depicted in Table 3 [ibuprofen, trolox and nordihydroguaiaretic (NDGA) are included for comparison].

**Figure 1:**
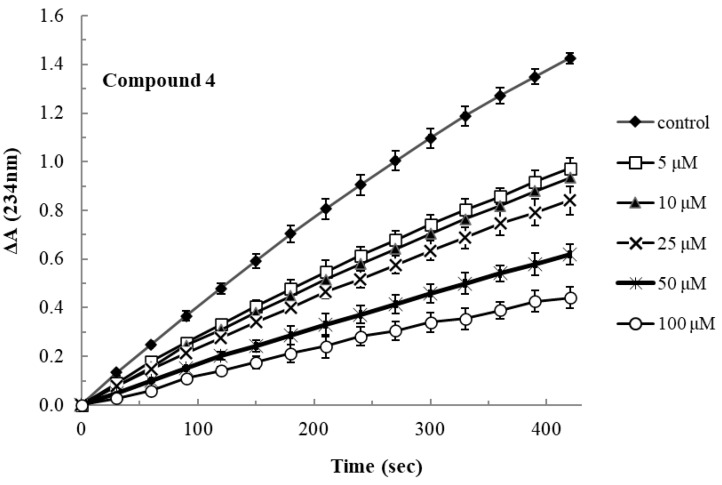
Lipoxygenase inhibitory activity of compound **4** at various concentrations over the incubation period.

LOX inhibition at various concentrations during 7 min of incubation of the most active compound **4** is shown below (Figure 1).

Most compounds presented moderate activity, although better than ibuprofen. The esterification of the serine derivative (compound **3**) yielded better LOX inhibitors (**4** and **6**) than the parent compounds. Interestingly, compound **4** (IC_50_ 36 μΜ) was almost 5.5 and 4 times more active than ibuprofen (IC_50_ 200 μΜ) and compound **3**, respectively. The increased activity of compound **4**, in relation to its parent molecules, may be explained by their increased lipophilicity (Table 4) and their molecular flexibility, which allows their insertion and occupation of the lipophilic channel of the lipoxygenase active site. The more rigid structures, like thiomorpholine and structure F of compounds **2** and **6**, may not allow such an interaction. The usage of higher than the saturating substrate concentrations of linoleic acid resulted in no inhibitory activity of the compounds, which may be an indication of competitive activity of the compounds with the substrate.

#### 2.3.2. Antioxidant Potential of the Compounds

In view of the pleiotropic effects of ROS and RNS on the progression of inflammation and the neural and cardiovascular degenerative disorders, inducing also lipotoxicity [67,68], the compounds were tested for their ability to scavenge the stable DPPH free radical and their ability to inhibit the peroxidation of lipids, induced by ferrous ions, which is regenerated with the electron-donating effect of ascorbic acid. Compounds **1**–**4** had minor or no antioxidant activity. The per cent interaction (mean values) with DPPH and the IC_50_ values on lipid peroxidation of the active compounds **5** and **6** and trolox (compound **11**, Scheme 2) are shown in Table 5.

Compound **5** had pronounced activity, being ten-fold stronger inhibitor of lipid peroxidation than trolox, with similar scavenging activity to that of trolox. The antioxidant activity is related to the phenolic hydrogen abstraction, leaving a phenoxyl radical. The radical is stabilized by enhanced conjugation on the chromane ring (compound **5**) or the cinnamic structure (compound **6**). The antioxidant effect of compound **5** is also related to the two-electron-donating effect of the chromane moiety. The increased lipophilicity of the compounds (Table 4) enables them to approach the lipid phase where peroxidation takes place. Thus, the relatively increased activity of our compounds in comparison to trolox may in part be related to their lipophilicity, together with their radical scavenging activity. 

**Figure 2:**
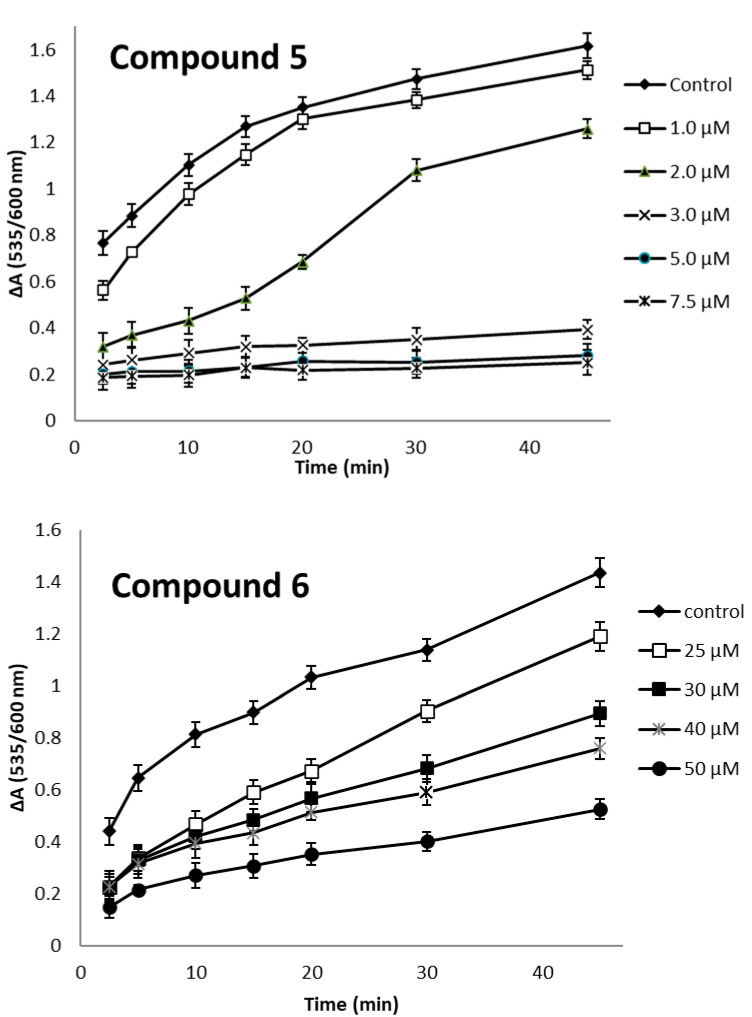
Concentration and time progression of lipid peroxidation affected by compounds **5** and **6**.

The time course of lipid peroxidation, as affected by various concentrations of the active compounds **5** and **6** is shown in Figure 2.

## 3. Materials and Methods

Commercially available chemicals and enzymes were of the appropriate purity and purchased from Merck (Kenilworth, NJ, USA) or Sigma (St. Louis, MO, USA). A Perkin Elmer Spectrum BX FT-IR spectrometer (Waltham, MA, USA) was used for IR spectra. BRUKER Avance III-300 MHz or AGILENT DD2-500 MHz spectrometers (Santa Clara, CA, USA) were used for ^1^H NMR and ^13^C NMR spectroscopy [shifts *δ* (ppm), s: singlet; d: doublet; t: triplet; m: multiplet]. MEL-TEMPII (Laboratory Devices, Sigma-Aldrich, Milwaukee, WI, USA) was used for the determination of the melting points (mp), and a Perkin-Elmer 2400 CHN elemental analyzer (Waltham, MA, USA) for the microanalyses. Thin-layer chromatography (TLC silica gel 60 F254 aluminum sheets, Merck, Kenilworth, NJ, USA) was applied to monitor the progress of the reactions. For the in vivo experiments, Wistar rats (160–220 g, 3–4 months old) were kept in the Centre of the School of Veterinary Medicine (EL54 BIO42), Aristotelian University of Thessaloniki, which is registered by the official state veterinary authorities, and all procedures were performed in harmonization with the European Directive 2010/63/EEC. The experimental protocols were approved by the Animal Ethics Committee of the Prefecture of Central Macedonia (no. 270073/2499 and 270079/2500).

### 3.1. Synthesis

#### 3.1.1. Procedure for the Synthesis of Compounds **1**–**3**

To a stirred solution of amines **7** or **8** (1.2 mmol) or a suspension of hydrochloride **9** (1.3 mmol) in CH_2_Cl_2_ (10 mL) at 0–5 °C, a solution of 2-propylpentanoyl chloride (1 mmol) in CH_2_Cl_2_ (10 mL) and Et_3_N (1.1 mmol for **7** and **8** and 2.6 mmol for **9**) were added dropwise at 0–5 °C. After 15 min at 0–5 °C, stirring continued for 4 h at r.t. The mixture was washed with deionized water (20 mL) and a 5% solution of NaHCO_3_ (20 mL) and dried over Na_2_SO_4_, and the solvent was removed under reduced pressure. The acquired crude material was purified by flash column chromatography, with EtOAc/petroleum ether 1:2 and then 1:1, to give compound **1** (170 mg, 76% yield) as a white solid (m.p. 160 °C), EtOAc/petroleum ether 1:8 to give compound **2** (197 mg, 86% yield) as a clear oil, and EtOAc/petroleum ether 1:2 to give compound **3** (201 mg, 82% yield) as a white solid (m.p. 93–94 °C).

#### 3.1.2. Procedure for the Synthesis of Compounds **4**–**6**

To a stirred solution of compound **3** (1.1 mmol) in CH_2_Cl_2_ (10 mL), solutions of the corresponding acids, compounds **10**, **11** or **12** (1mmol), N,N-dimethylaminopyridine (DMAP, 0.1 mmol) and N,N′-dicyclohexylcarbodiimide (DCC, 1.3 mmol) in 10 mL CH_2_Cl_2_ were added successively, and the mixture was stirred for 12 h at r.t. The acquired crude material was purified by flash column chromatography, with EtOAc/petroleum ether 1:5 to give compound **4** (325 mg, 78% yield) as a white solid (m.p. 68–71 °C), EtOAc/petroleum ether 1:3 to give compound **5** (267 mg, 56% yield) as a white solid (m.p. 108–120 °C) and EtOAc/petroleum ether 1:2 to give compound **6** (418 mg, 83% yield) as a clear red oil.

**2-Propyl-N-(pyridin-4-yl)pentanamide, 1.**

IR (Nujol) λ_max_: 3306, 3228 (N-H), 1702 (C=O), 1599 (C-C aromatic) cm^−1^ (please see Supplementary Materials document). ^1^H NMR (CDCl_3_), *δ* (ppm): 0.90 [t, 6H, *J* = 7.3 Hz, (C**H_3_**-CH_2_-CH_2_)_2_CH-], 1.27–1.39 [m, 4H, (CH_3_-C**H_2_**-CH_2_)_2_CH-], 1.42–1.52 and 1.64–1.72 [m, 4H, (CH_3_-CH_2_-C**H_2_**)_2_CH-], 2.28 [tt, 1H, *J* = 10.0, 5.2 Hz, (CH_3_-CH_2_-CH_2_)_2_C**H**-], 7.56 [d, 2H, *J* = 6.4 Hz, pyridine C3, C5 H], 8.15 [s, 1H, NH], 8.46 [d, 2H, *J* = 6.4 Hz, pyridine C2, C6 H]. ^13^C NMR (CDCl_3_), *δ* (ppm): 14.09 [2C, (**C**H_3_-CH_2_-CH_2_)_2_CH-], 20.80 [2C, (CH_3_-**C**H_2_-CH_2_)_2_CH-], 35.15 [2C, (CH_3_-CH_2_-**C**H_2_)_2_CH-], 48.70 [1C, (CH_3_-CH_2_-CH_2_)_2_**C**H-], 113.70 [2C, pyridine **C3**, **C5**], 145.46 [1C, pyridine **C4**], 150.24 [2C, pyridine **C2**, **C6**], 175.72 [1C, N-**C**=O]. Anal. Calcd for C_13_H_20_Ν_2_Ox0.1H_2_O: C, 69.73; H, 9.18; Ν, 12.51. Found: C, 69.96; H, 9.35; Ν, 12.79.

**2-Propyl-1-thiomorpholinopentan-1-one, 2.**

IR λ_max_: 2956, 2929, 2872 (C-H alkyl), 1640 (C=O amide) cm^−1^. ^1^H NMR (CDCl_3_), *δ* (ppm): 0.91 [t, 6H, *J* = 7.1 Hz, (C**H_3_**-CH_2_-CH_2_)_2_CH-], 1.18–1.34 [m, 4H, (CH_3_-C**H_2_**-CH_2_)_2_CH-], 1.34–1.49 [m, 2H, (CH_3_-CH_2_-C**H_2_**)_2_CH-], 1.60–1.74 [m, 2H, (CH_3_-CH_2_-C**H_2_**)_2_CH-], 2.52–2.71 [m, 5H, -C**H_2_**-S-C**H_2_**- and (CH_3_-CH_2_-CH_2_)_2_C**H**-], 3.82–3.97 [m, 4H, -C**H_2_**-N-C**H_2_**-]. ^13^C NMR (CDCl_3_), *δ* (ppm): 14.22 [2C, (**C**H_3_-CH_2_-CH_2_)_2_CH-], 20.89 [2C, (CH_3_-**C**H_2_-CH_2_)_2_CH-], 28.20, 27.74 [2C, N-CH_2_**C**H_2_S], 35.29 [2C, (CH_3_-CH_2_-**C**H_2_)_2_CH-], 40.58 [1C, (CH_3_-CH_2_-CH_2_)_2_**C**H-], 48.35, 44.53 [2C, N-**C**H_2_CH_2_S], 174.81 [1C, N-**C**=O]. Anal. Calcd for C_12_H_23_ΝO_5_: C, 62.83; H, 10.11; Ν, 6.11. Found: C, 62.87; H, 10.21; Ν, 6.29.

**Methyl 3-hydroxy-2-(2-propylpentanamido)propanoate, 3.**

IR (Nujol) λ_max_: 3546 (O-H), 3289 (N-H), 1737 (O-**C=O**), 1641 (N-**C=O**) cm^−1^. ^1^H NMR (CDCl_3_), *δ* (ppm): 0.90 [t, 6H, *J* = 7.2 Hz, (C**H_3_**-CH_2_-CH_2_)_2_CH-], 1.30 [tq, 4H, *J* = 20.7, 7.2 Hz, (CH_3_-C**H_2_**-CH_2_)_2_CH-], 1.36–1.46 [m, 2H, (CH_3_-CH_2_-C**H_2_**)_2_CH-], 1.53–1.68 [m, 2H, (CH_3_-CH_2_-C**H_2_**)_2_CH-], 2.12–2.21 [m, 1H, (CH_3_-CH_2_-CH_2_)_2_C**H**-], 2.40 [s, 1H, -CH-CH_2_-O**H**], 3.79 [bs, 3H, -O-C**H_3_**], 3.94 [ddd, 2H, *J* = 14.4, 11.1, 3.7 Hz, -CH-C**H_2_**-OH], 4.64–4.73 [m, 1H, -C**H**-CH_2_-OH], 6.45 [d, 1H, *J* = 6.2 Hz, -NH-]. ^13^C NMR (CDCl_3_), *δ* (ppm): 14.06 [2C, (**C**H_3_-CH_2_-CH_2_)_2_CH-], 20.74, 20.66 [2C, (CH_3_-**C**H_2_-CH_2_)_2_CH-], 35.12 [2C, (CH_3_-CH_2_-**C**H_2_)_2_CH-], 47.41 [1C, (CH_3_-CH_2_-CH_2_)_2_**C**H-], 52.74 [1C, COO**C**H_3_], 54.56 [1C, NH-**C**H-CH_2_-OH], 63.91 [1C, NH-CH-**C**H_2_-OH], 170.98 [1C, O-**C**=O], 176.69 [1C, N-**C**=O]. Anal. Calcd for C_12_H_23_NO_4_: C, 58.75; H, 9.45; N, 5.71. Found: C, 58.99; H, 9.78; N, 5.74.

**Methyl 3-((2-(4-isobutylphenyl)propanoyl)oxy)-2-(2-propylpentanamido)propanoate, 4.**

IR (Nujol) λ_max_: 3319 (N-H), 1732 (O-**C=O**), 1639 (N-**C=O**), 1541 (C-C aromatic) cm^−1^. ^1^H NMR (CDCl3), *δ* (ppm): 0.82–0.91 [m, 12H, (C**H_3_**-CH_2_-CH_2_)_2_CH- and (C**H_3_**)**_2_**-CH-CH_2_], 1.18–1.38 [m, 4H, (CH_3_-C**H_2_**-CH2)_2_CH-], 1.47 [3H, dd, *J* = 7.2, 1.8 Hz, **C**H_3_-CHC=O], 1.45–1.58 [m, 2H, (CH_3_-CH_2_-C**H_2_**)_2_CH-], 1.79–1.87 [m, 1H, (CH_3_-CH_2_-CH_2_)_2_C**H**-], 1.90–2.06 [m, 1H, (CH_3_)**_2_**-C**H**-CH_2_], 2.44 [d, 2H, *J* = 7.2 Hz, (CH_3_)**_2_**-CH-C**H_2_**], 3.69, 3.60 [s, 3H, -O-C**H_3_**], 3.64–3.71 [m, 1H, CH_3_-**C**HC=O], 4.30–4.46 [m, 2H, -CH-C**H_2_**-O-], 4.80–4.85 [m, 1H, -C**H**-CH_2_-O-], 6.06, 5.89 [1H, d, *J* = 7.6 Hz, -NH-], 7.08–7.18 [m, 4H, aromatic]. ^13^C NMR (CDCl_3_), *δ* (ppm): 14.07 [2C, (**C**H_3_-CH_2_-CH_2_)_2_CH-], 18.15, 17.85 [1C, **C**H_3_-CH-C=O], 20.61, 20.56 [2C, (CH_3_-**C**H_2_-CH_2_)_2_CH-], 22.34, 22.32 [2C, (**C**H_3_)_2_-CH-CH_2_], 30.16 [1C, (CH_3_)_2_-**C**H-CH_2_], 35.08, 35.04 [2C, (CH_3_-CH_2_-**C**H_2_)_2_CH-], 44.83 [1C, CH_3_-**C**H-C=O], 44.98 [1C, (CH_3_)_2_-CH-**C**H_2_], 47.32, 47.29 [1C, CH_3_-**C**H-C=O], 51.40, 51.34 [1C, COO**C**H_3_], 52.65, 52.57 [1C, NH-**C**H-CH_2_-OH], 64.26, 64.19 [1C, NH-CH-**C**H_2_-OH], 127.14, 127.13 [2C, aromatic **C2**, **C6**], 129.38, 129.34 [2C, aromatic **C3**, **C5**], 137.26, 137.25 [1C, aromatic **C4**], 140.79, 140.72 [1C, aromatic **C4**], 169.96, 169.90 [1C, CH_3_O-**C**=O], 174.09, 174.07 [1C, CH_3_-C-**C**=O], 175.70, 175.66 [1C, N-**C**=O]. Anal. Calcd for C_25_H_39_ΝO_5_x0.1H_2_O: C, 69.25; H, 9.07; Ν, 3.23. Found: C, 69.35; H, 8.98; Ν, 3.21.

**3-Methoxy-3-oxo-2-(2-propylpentanamido)propyl 6-hydroxy-2,5,7,8-tetramethylchroman-2-carboxylate, 5.**

IR (Nujol) λ_max_: 3531 (O-H), 3268 (N-H), 1750, 1742 (O-**C=O** LL and LD), 1652 (N-**C=O**) cm^−1^. ^1^H NMR (CDCl_3_), *δ* (ppm): 0.84–0.96 [m, 6H, (C**H_3_**-CH_2_-CH_2_)_2_CH-], 1.05–1.58 [m, 8H, (CH_3_-C**H_2_**-CH_2_)_2_CH- and (CH_3_-CH_2_-C**H_2_**)_2_CH-], 1.62 and 1.60 [s, 3H, -C**H_3_** C1 chromane], 1.76–1.97 [m, 2H, C2 chromane], 2.21, 2.20, 2.10 and 2.09 [s, 9H, aromatic -CH_3_], 2.38–2.75 [m, 3H, C3 chromane and (CH_3_-CH_2_-CH_2_)_2_C**H**-], 3.76 and 3.60 [s, 3H, -O-CH_3_], 4.24–4.32 [m, 1H, -NH-CH-C**H_2_**-], 4.59 [dd, 1H, *J* = 11.3, 2.9 Hz, -NH-CH-C**H_2_**-], 4.80–4.87 [m, 0,5H, -NH-C**H**-CH_2_-], 4.93 [dt, 0,5H, *J* = 7.8, 2.9 Hz, -NH-C**H**-CH_2_-], 5.54 and 5.51 [s, 1H, phenol -OH], 5.89 [d, 1H, *J* = 7.8 Hz, -NH-]. ^13^C NMR (CDCl_3_), *δ* (ppm): 11.28, 11.22 [1C, chromane C7-**C**H_3_], 11.80, 11.73 [1C, chromane C8-**C**H_3_], 12.18, 12.23 [1C, chromane C5-**C**H_3_], 14.20, 14.13 [2C, (**C**H_3_-CH_2_-CH_2_)_2_CH-], 20.86, 20.80, 20.66, 20.58, 20.53, 20.48 [2C, (CH_3_-**C**H_2_-CH_2_)_2_CH-], 25.69 [1C, chromane **C4**], 25.79 [1C, chromane C2-**C**H_3_], 30.57, 30.48 [1C, chromane **C3**], 35.20, 35.18, 35.12, 35.09 [2C, (CH_3_-CH_2_-**C**H_2_)_2_CH-], 47.41, 47.39 [1C, -CH_2_-CH_2_)_2_**C**H-], 51.13, 50.44 [1C, COO**C**H_3_], 52.66, 52.49 [1C, NH-**C**H-CH_2_-OH], 65.19, 64.62 [1C, NH-CH-**C**H_2_-OH], 77.10 [1C, chromane **C3**], 145.48, 122.49, 122.31, 121.18, 118.41, 116.82 [6C, aromatic C], 169.60, 169.57 [1C, CH_3_O-**C**=O], 173.83, 173.70 [1C, O-C-**C**=O], 175.66 [1C, N-**C**=O]. Anal. Calcd for C_26_H_39_ΝO_7_: C, 65.39; H, 8.23; Ν, 2.93. Found: C, 65.52; H, 8.11; Ν, 2.80.

**(E)-3-methoxy-3-oxo-2-(2-propylpentanamido)propyl 3-(3,5-di-tert-butyl-4-hydroxyphenyl)acrylate, 6.**

IR λ_max_: 3630, 3581 (O-H), 3324 (N-H), 3069 (C-H aromatic), 2936, 2873 (C-H alkyl), 1742, 1717 (O-**C=O** ester), 1660, 1630 (N-**C=O**), 1596 (C-C aromatic) cm^−1^. ^1^H NMR (CDCl_3_), *δ* (ppm): 0.91 [dd, 6H, *J* = 14.6, 7.1 Hz, (C**H_3_**-CH_2_-CH_2_)_2_CH-], 1.24–1.41 [m, 6H, (CH_3_-C**H_2_**-CH_2_)_2_CH- and (CH_3_-CH_2_-C**H_2_**)_2_CH-], 1.48 [s, 12H, -CH_3_ trans], 1.55–1.65 [m, 2H, (CH_3_-CH_2_-C**H_2_**)_2_CH-], 2.18 [s, 6H, -CH_3_ cis], 2.25–2.35 [m, 1H, (CH_3_-CH_2_-CH_2_)_2_C**H**-], 3.81 [s, 3H, -O-CH_3_], 4.56 [dd, 2H, *J* = 11.4, 4.1 Hz, -NH-CH-C**H_2_**-], 4.97 [dt, 1H, *J* = 8.0, 4.1 Hz, -NH-C**H**-CH_2_-], 5.55 [s, 1H, phenol -OH], 6.26 [d, 1H, *J* = 15.9 Hz, -C**H**=CH-C=O], 6.31 [s, 1H, -NH-], 7.38 [s, 2H, phenyl H], 7.66 [d, 1H, *J* = 15.9 Hz, -CH-C**H**-C=O]. ^13^C NMR (CDCl_3_), *δ* (ppm): 14.11, 14.08 [2C, (**C**H_3_-CH_2_-CH_2_)_2_CH-], 20.67, 20.66 [2C, (CH_3_-**C**H_2_-CH_2_)_2_CH-], 30.13 [6C, -**C**(CH_3_)_3_], 34.32 [2C, (CH_3_-CH_2_-**C**H_2_)_2_CH-], 35.16, 35.13 [2C, -**C**(CH_3_)_3_], 47.38 [1C, -CH_2_-CH_2_)_2_**C**H-], 51.64 [1C, COO**C**H_3_], 52.79 [1C, NH-**C**H-CH_2_-OH], 63.66 [1C, NH-CH-**C**H_2_-OH], 113.26 [1C, CH=**C**H-C=O], 125.46 [1C, **C**-CH=CH-C=O], 125.61 [2C, phenyl **C2**, **C6**], 136.41 [2C, phenyl **C3**, **C5**], 147.10 [1C, **C**H=CH-C=O], 156.43 [1C, phenyl **C4**], 167.11 [1C, CH=CH-**C**=O], 170.27 [1C, CH_3_O-**C**=O], 175.87 [1C, N-**C**=O]. Anal. Calcd for C_29_H_45_ΝO_6_: C, 69.15; H, 9.01; Ν, 2.78. Found: C, 68.98; H, 9.16; Ν, 2.90.

### 3.2. Biological Experiments

#### 3.2.1. Carrageenan-Induced Paw Edema

An aqueous solution (1% *w*/*v*) of carrageenan and solutions of the compounds (0.15 mmol/kg, in saline with a few drops of Tween 80) were prepared. Each test group of 6 rats was administered the compounds at a dose of 0.15 mmol/kg *i.p.* After 5 min, carrageenan (0.1 mL) was given *i.d*. into the hind paw of the treated and the control groups, and 3.5 h later, the produced weight difference between the hind paws was estimated as per cent increase compared to the carrageenan control group [27].

#### 3.2.2. Effect on Plasma Lipids

Male rats (180–220 g b.w.) were divided into seven groups, one group serving as the hyperlipidemic control. All rats were given *i.p.* Triton WR 1339 in saline (200 mg/kg). After 1 h, the treatment groups received the compounds (0.15 mmol/kg, *i.p*.) in saline with drops of Tween 80, while the control group received the liquid vehicle. Twenty-four hours later, blood of all rats was collected from the aorta under sodium hexobarbital anesthesia, in heparinized tubes, centrifuged (3000 rpm, 20 min), and the plasma concentration of total cholesterol, LDL-cholesterol and triglycerides was determined, using commercial kits [28,56,58].

#### 3.2.3. In Vitro Evaluation of Lipoxygenase Activity

For this experiment, Tris/HCl buffer solution 0.1 M, pH 9.0, 0.9% NaCl solution, 3 mM sodium linoleate solution (in Tris/HCl buffer), soybean lipoxygenase (2500 u/mL in 0.9% NaCl solution) and compounds solution (9 mΜ in absolute ethanol, analytical grade, iron content < 10^−5^% *w*/*v*) were prepared. The reaction mixture contained 2.6 mL of buffer, 0.2 mL of the enzyme solution and 0.1 mL of the compound solution. The reaction was initiated with the addition of 0.1 mL of sodium linoleate. The reaction was followed for 7 min, 28 °C, recording the absorbance every 30 s at 234 nm. Nordihydroguaiaretic acid was used as a reference. Sodium linoleate at 1 mM final concentration (higher than the saturating substrate concentration) was used for the estimation of the type of inhibition [27,58,69].

#### 3.2.4. In Vitro Lipid Peroxidation Evaluation as TBA-Reactive Material

Livers from untreated rats were homogenized in saline, centrifuged (9000× *g*, 20 min, 4 °C), and the supernatant was centrifuged (110,000× *g*, 40 min, 4 °C). The pellet was washed with saline by centrifugation (110,000× *g*, 40 min, 4 °C), resuspended in Tris/HCl buffer (pH 7.4), corresponding to 0.5 g liver/mL, and stored at −80 °C until use.

For the lipid peroxidation experiments, the microsomal suspension was inactivated by heating (90 °C, for 90 s), homogenized and used at a final concentration corresponding to 0.125 g liver per ml, or 4 mM fatty acid residues.

The incubation mixture contained heat-inactivated microsomal fraction, the tested compounds (in dimethylsulfoxide, at the appropriate concentrations) and the ascorbic acid/Fe^+2^ system as electron source. The control mixture contained dimethylsulfoxide in place of the compounds. The mixtures were incubated (37 °C) with shaking under air. Samples were taken at various time periods for a total of 45 min. The peroxidation reaction in each aliquot of the incubation (0.3 mL) was stopped by mixing with ice-cold 2-thiobarbituric acid (TBA)/trichloroacetic acid/HCl/butylated hydroxytoluene (BHT) solution (2 mL). The TBA/trichloroacetic acid/HCl solution was prepared by dissolving 41.6 mg TBA in l0 mL trichloroacetic acid (16.8% *w*/*v* in 0.125 N HCl). To a 10 mL TBA/trichloroacetic acid/HCl mixture, 1 mL BHT (1.5 mg/mL ethanol) was added. After heating (20 min, 90 °C) and centrifugation (3000 rpm, 15 min), the absorbance at 535 nm vs. 600 nm was recorded [27].

#### 3.2.5. In Vitro Interaction with the Stable Radical 1,1-Diphenyl-2-picrylhydrazyl (DPPH)

DPPH (2,2-diphenyl-1-picrylhydrazyl) radical scavenging assay is based on the transfer of one hydrogen atom from an antioxidant to the stable nitrogen-centered free radical DPPH•, leading to the conversion of the violet color of the radical to the reduced DPPH yellow compound. Ethanolic solution of DPPH• (400 μΜ) and appropriate concentrations of the examined compounds were prepared. The reaction mixture, containing DPPH• (200 μΜ) and the compounds under examination (200–25 μΜ) or ethanol (control), was incubated for 30 min at ambient temperature. Absorbance was recorded at 517 nm [11,70]. The percentage of DPPH• scavenging activity of the compounds was calculated via the equation:

% DPPH• reduction = [(Acontrol − Asample)/Acontrol] × 100(1)

## 4. Conclusions

In summary, VA inhibits the production of inflammatory factors and the migration of immune cells, offering an anti-inflammatory effect, with parallel cell and tissue damage attenuation. Therefore, the acute inflammation inhibitory effect, the hypolipidemic potency, the protection against lipid peroxidation and the antioxidant effect of the conjugated VA derivatives in this report seem to add towards the multi-functional characteristics of VA acid. Thus, the lipoxygenase inhibition of the compounds, the antioxidant effect of trolox and (*E*)-3-(3,5-di-tert-butyl-4-hydroxyphenyl)acrylic acid, the lipid lowering potential of thiomorpholine and the increased anti-inflammatory and hypolipidemic effect of 4-aminopyridine, as integrated parts in the examined compounds, may serve as lead to VA derivatives against inflammatory disorders that need a multi-targeted approach, offering also reduction in drug interactions. Future research would be based on issues concerning the pharmacological characteristics of these compounds and the potential changes in their cores that could elucidate the full array of activities of such pleiotropically acting derivatives.

## Figures and Tables

**Table 1:** Effect of the tested compounds (0.15 mmol/kg of body weight *i.p*.) on paw edema produced by carrageenan administration in rats ^a^.

Compound	% Edema Reduction
**1**	48 **
**2**	37 *
**3**	29 *
**4**	45 **
**5**	46 **
**6**	30 *
**Ibuprofen**	36 **

^a^ The effect is expressed as percent inhibition of edema in comparison to carrageenan controls. Each value is the mean obtained from six rats. Significant difference from control: * *p* < 0.05, ** *p* < 0.005 (Student’s *t* test, unpaired).

**Table 2:** Antihyperlipidemic activity of the compounds.

Compound	% Reduction			
	Dose (*i.p*.) (μmol/kg)	TC	TG	LDL-C
**1**	150	50.2 ***	52.0 ***	76.3 **
**2**	150	71.1 **	52.8 *	79.1 **
**3**	150	57.0 ***	33.7 *	33.2 *
**4**	150	61.4 ***	38.9 *	58.2 **
**5**	150	65.9 **	52.3 ***	59.2 *
**6**	150	48.9 *	21.0 *	44.8 *
**Simvastatin**	150	73.0 ***	-	70.0 ***
**Ibuprofen**	300	41.0 ***	38.0 ***	41.6 ***

Values are obtained from 6 animals. Significant difference from hyperlipidemic control group: * *p* < 0.02, ** *p* < 0.005, *** *p* < 0.001 (Student’s *t* test, unpaired). -: not statistically significant result.

**Table 3:** Inhibitory activity of the tested compounds (concentration 100 μM) against lipoxygenase.

Compound	% Inhibition
**1**	48
**2**	27
**3**	20
**4**	69
**5**	23
**6**	30
**Ibuprofen**	12
**Trolox**	-
**NDGA**	94

-: inactive. All tests were carried out three times. Standard deviation (SD) was less than 10% of the mean value.

**Table 4:** Physicochemical properties of the synthesized compounds ^a^.

Compound	Molecular Weight	Total Polar Surface Area (Å^2^)	H Bond Donors	H Bond Acceptors	ClogP	Lipinsky’s Rule of 5 Violations
**1**	220.31	41.99	1	3	3.45	0
**2**	229.38	20.31	0	2	2.88	0
**3**	243.34	66.40	2	4	1.99	0
**4**	433.58	81.71	1	6	6.77	1
**5**	477.59	111.17	2	8	5.69	1
**6**	503.67	101.94	2	7	7.39	2

^a^ Calculated with ChemBioDraw Ultra 12.0, Clog*P* for Windows v. 4.0, BioByte Corp.

**Table 5:** Radical scavenging activity of compounds **5**, **6** and **trolox**, at various concentrations, against DPPH free radical (200 μΜ), after 30 min of incubation, and their inhibitory effect on lipid peroxidation (after 45 min of incubation).

Comp.	Percent Interaction with DPPH				Inhibition of Lipid Peroxidation IC_50_ (μΜ)
	200 μΜ	100 μΜ	50 μΜ	25 μΜ	
**5**	91	84	51	25	2.6 μΜ
**6**	90	59	26	15	45 μΜ
**Trolox**	92	90	38	22	25 μΜ

All determinations were performed in triplicate, and the standard deviation is always within ±10% of the mean value.

## Data Availability

The original contributions presented in this study are included in the article/Supplementary Materials. Further inquiries can be directed to the corresponding author.

## Supplementary Materials

The following supporting information can be downloaded at: https://www.mdpi.com/article/10.3390/molecules30112339/s1, Figure S1. 1H NMR (500 MHz, CDCl3) of compound **1**; Figure S2. 13C NMR (500 MHz, CDCl3) of compound **1**; Figure S3. IR spectrum (Nujol) of compound **1**; Figure S4. 1H NMR (300 MHz, CDCl3) of compound **2**; Figure S5. 13C NMR (500 MHz, CDCl3) of compound **2**; Figure S6. IR spectrum (KBr disc) of compound **2**; Figure S7. 1H NMR (300 MHz, CDCl3) of compound **3**; Figure S8. 13C NMR (500 MHz, CDCl3) of compound **3**; Figure S9. IR spectrum (Nujol) of compound **3**; Figure S10. 1H NMR (500 MHz, CDCl3) of compound **4**; Figure S11. 13C NMR (500 MHz, CDCl3) of compound **4**; Figure S12. IR spectrum (Nujol) of compound **4**; Figure S13. 1H NMR (300 MHz, CDCl3) of compound **5**; Figure S14. 13C NMR (500 MHz, CDCl3) of compound **5**; Figure S15. IR spectrum (Nujol) of compound **5**; Figure S16. 1H NMR (300 MHz, CDCl3) of compound **6**. Figure S17. 13C NMR (500 MHz, CDCl3) of compound **6**; Figure S18. IR spectrum (KBr disc) of compound **6.**
