# Supplementary material for: Multi-Targeting Valproic Acid Conjugates as Potent Agents Against Inflammation and Hyperlipidemia
Source: Molecules. 2025 May 27;30(11):2339. doi: 10.3390/molecules30112339 (PMC12156246; doi:10.3390/molecules30112339)
Supplement: Supplementary file 1 [file molecules-30-02339-s001.zip › molecules-3612127-supplementary.pdf]

Supporting Information

Multi-targeting valproic acid conjugates as potent agents against inflammation and hyperlipidemia

Panagiotis Theodosis-Nobelos[1,*], Eleni A. Rekka[2]

[1] Department of Pharmacy, School of Health Sciences, Frederick University, Nicosia, 1036, Cyprus.

[2] Department of Pharmaceutical Chemistry, School of Pharmacy, Aristotelian University of Thessaloniki, Thessaloniki 54124, Greece.

* Author for correspondence:

Panagiotis Theodosis-Nobelos

Tel.: (+357) 22394394

Fax: (+357) 22438234

E-mail: hsc.np@frederick.ac.cy

Contents

Figure S1. ^{1}H NMR (500 MHz, $CDCl_3$) of compound **1**. 3

Figure S2. ^{13}C NMR (500 MHz, $CDCl_3$) of compound **1**. 4

Figure S3. IR spectrum (Nujol) of compound **1**...5

Figure S4. ^{1}H NMR (300 MHz, $CDCl_3$) of compound **2**. 6

Figure S5. ^{13}C NMR (500 MHz, $CDCl_3$) of compound **2**. 7

Figure S6. IR spectrum (KBr disc) of compound **2**..8

Figure S7. ^{1}H NMR (300 MHz, $CDCl_3$) of compound **3**. 9

Figure S8. ^{13}C NMR (500 MHz, $CDCl_3$) of compound **3**. 10

Figure S9. IR spectrum (Nujol) of compound **3**.. 11

Figure S10. ^{1}H NMR (500 MHz, $CDCl_3$) of compound **4**. 12

Figure S11. ^{13}C NMR (500 MHz, $CDCl_3$) of compound **4**. 13

Figure S12. IR spectrum (Nujol) of compound **4**..14

Figure S13. ^{1}H NMR (300 MHz, $CDCl_3$) of compound **5**. 15

Figure S14. ^{13}C NMR (500 MHz, $CDCl_3$) of compound **5**. 16

Figure S15. IR spectrum (Nujol) of compound **5**..17

Figure S16. ^{1}H NMR (300 MHz, $CDCl_3$) of compound **6**. 18

Figure S17. ^{13}C NMR (500 MHz, $CDCl_3$) of compound **6**. 19

Figure S18. IR spectrum (KBr disc) of compound **6**...20

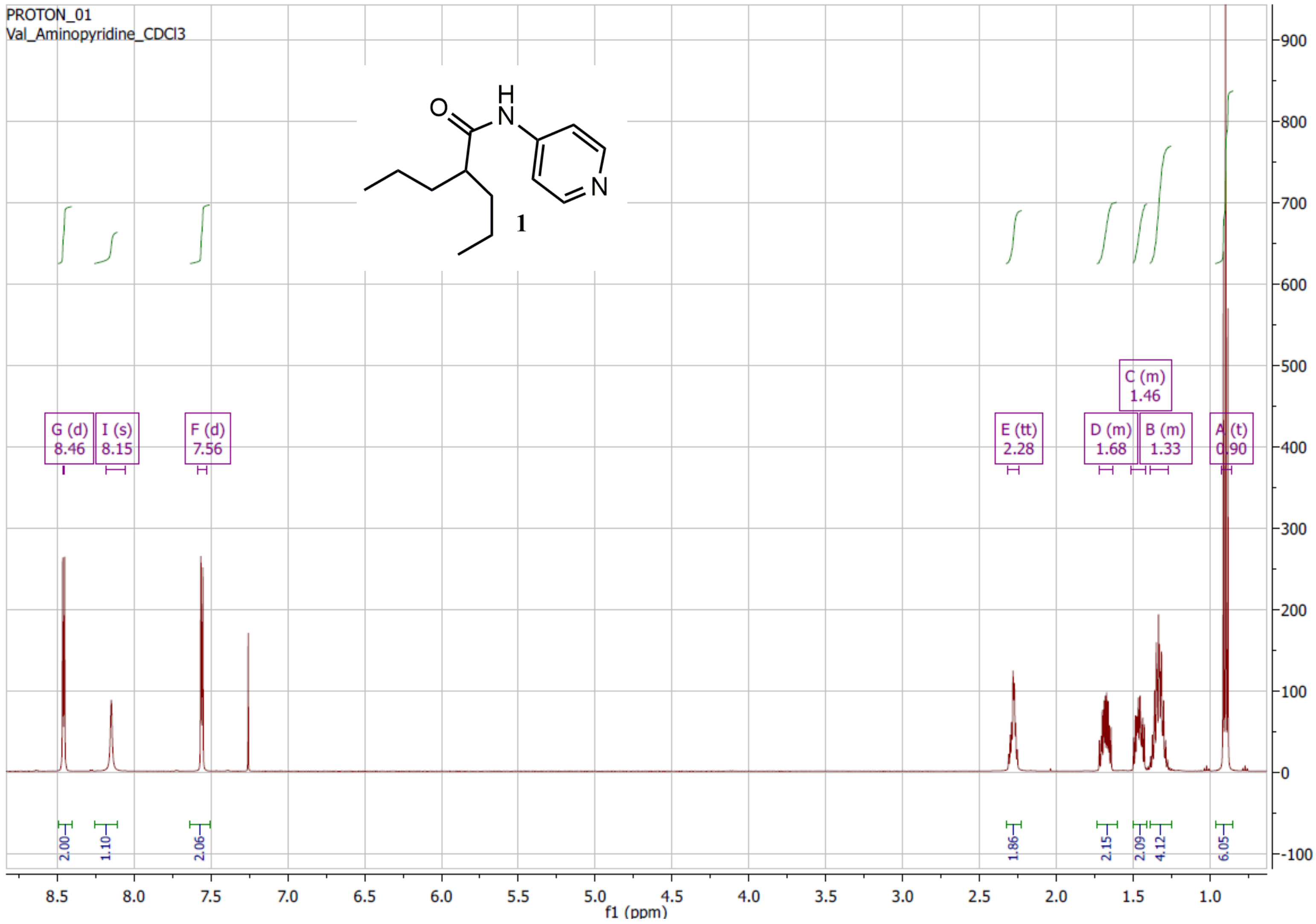

Figure S1. ^{1}H NMR (500 MHz, $CDCl_3$) of compound **1**.

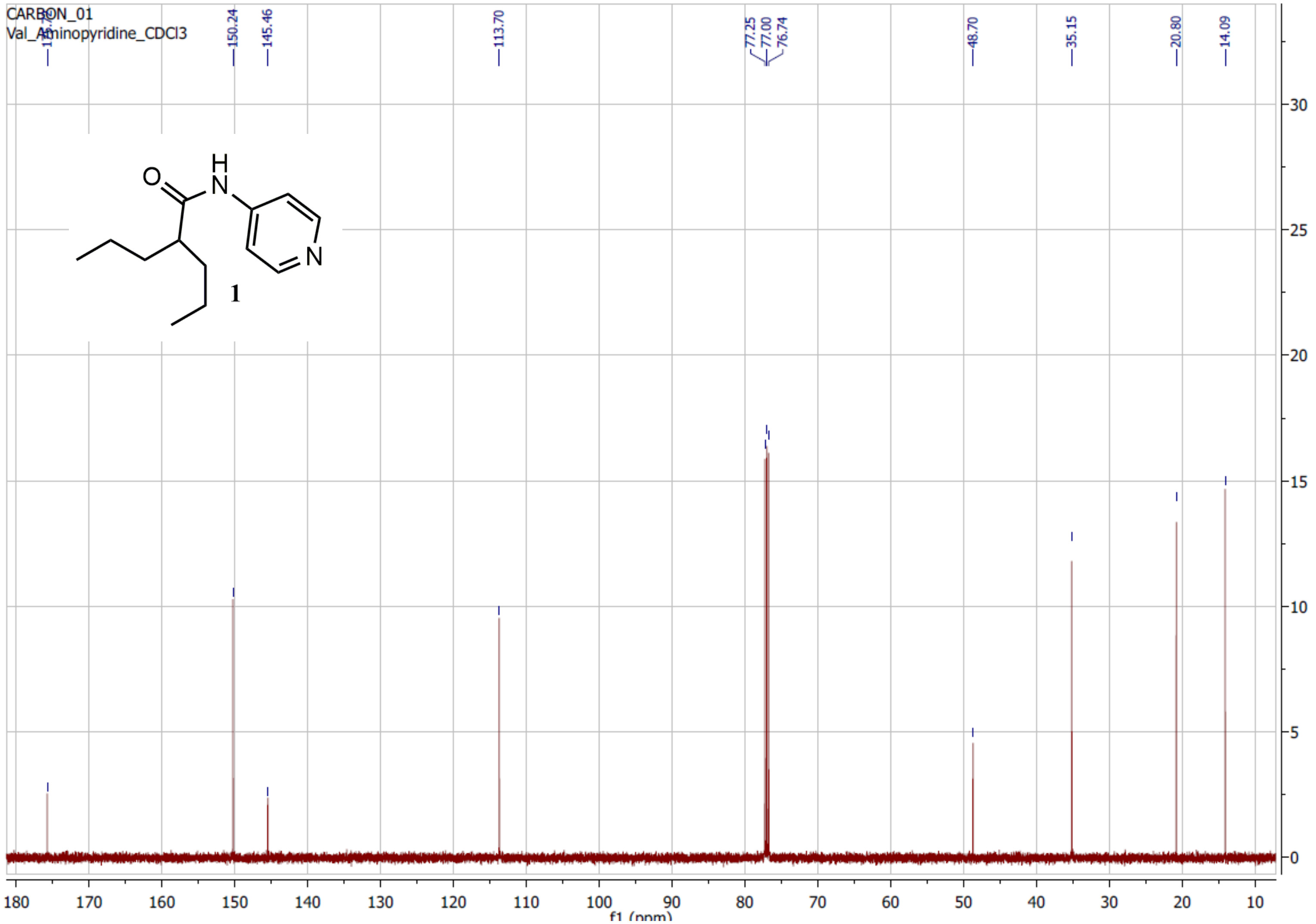

Figure S2. ^{13}C NMR (500 MHz, $CDCl_3$) of compound **1**.

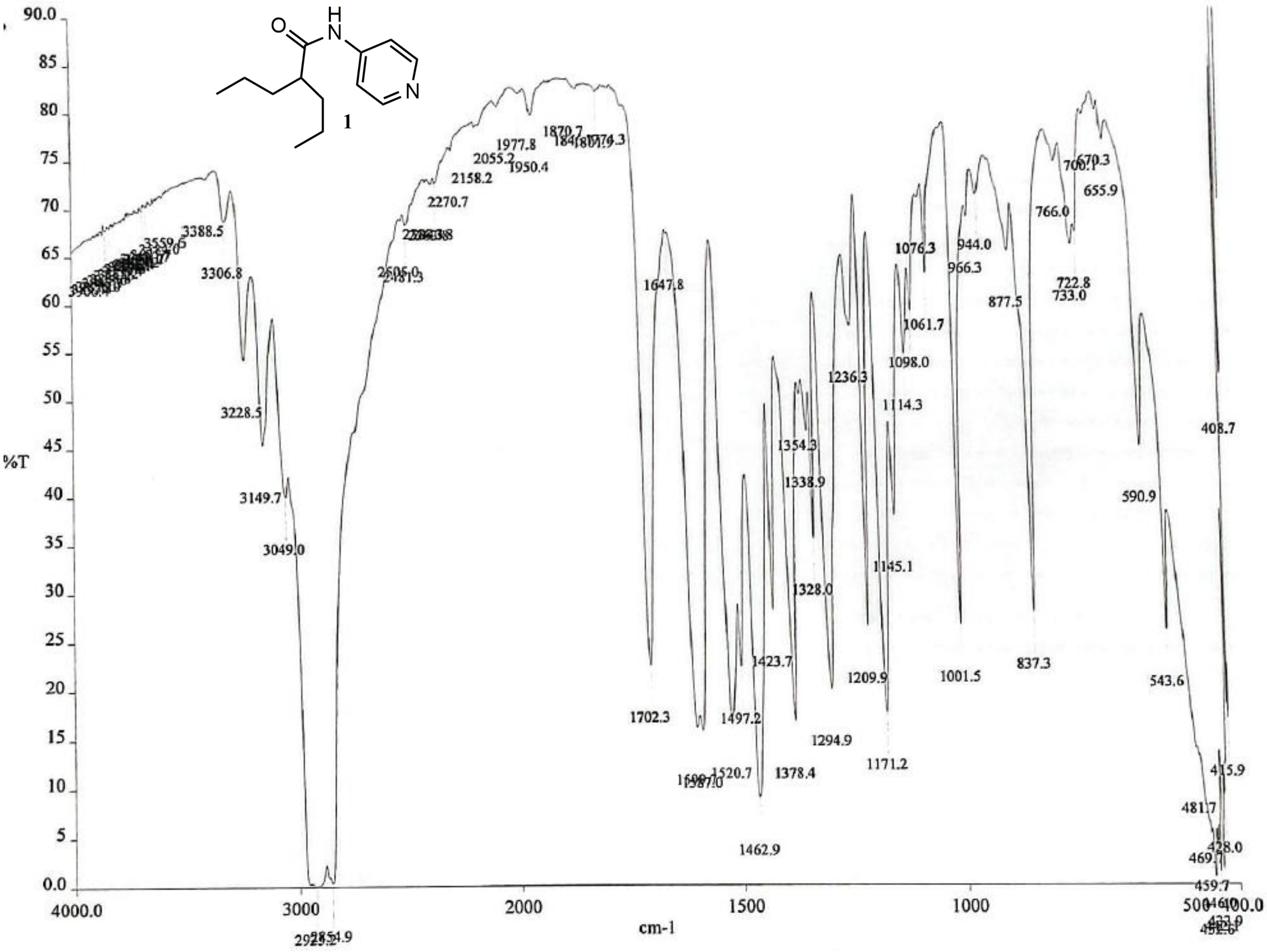

Figure S3. IR spectrum (Nujol) of compound **1**.

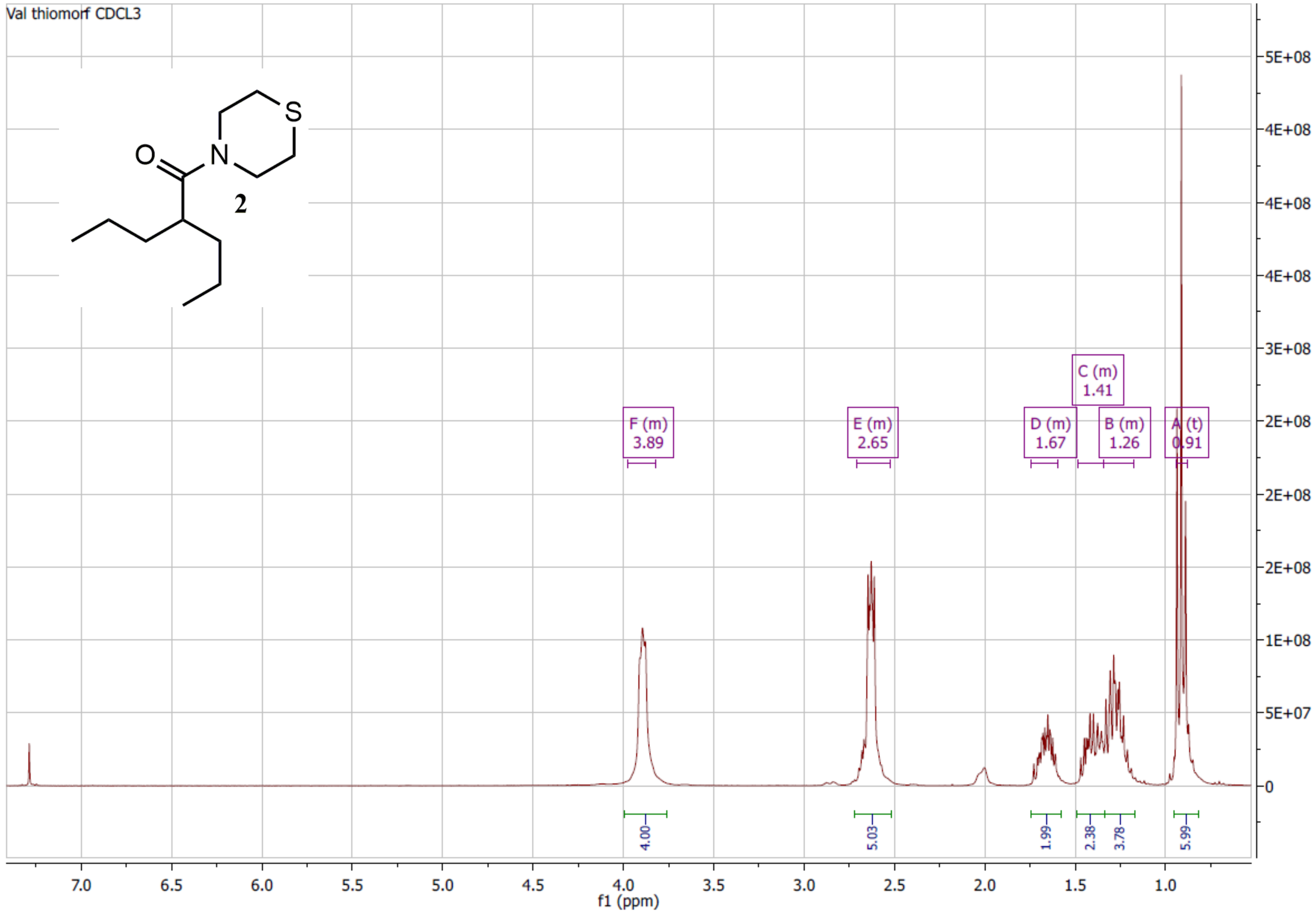

Figure S4. ^{1}H NMR (300 MHz, $CDCl_3$) of compound **2**.

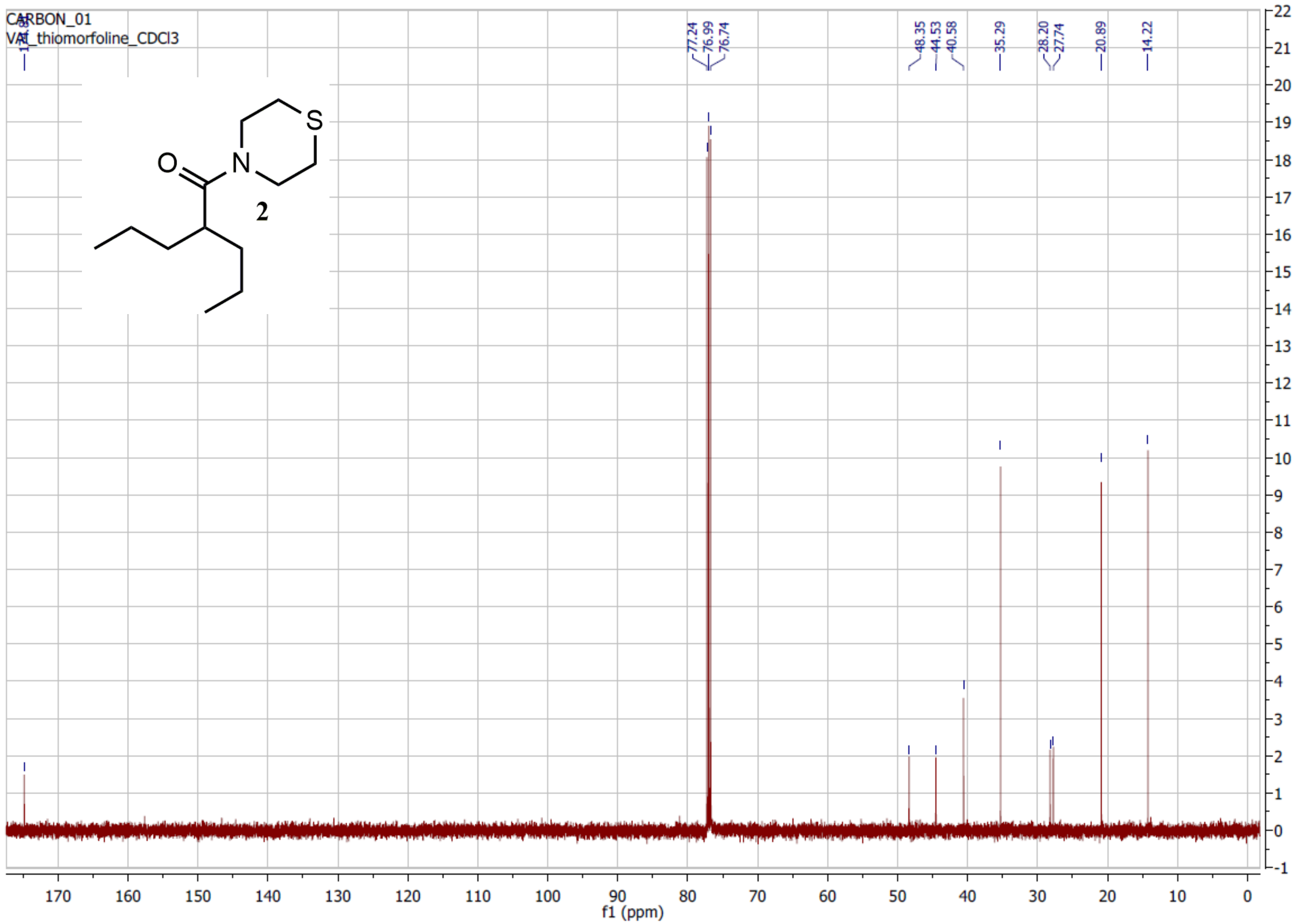

Figure S5. ^{13}C NMR (500 MHz, $CDCl_3$) of compound **2**.

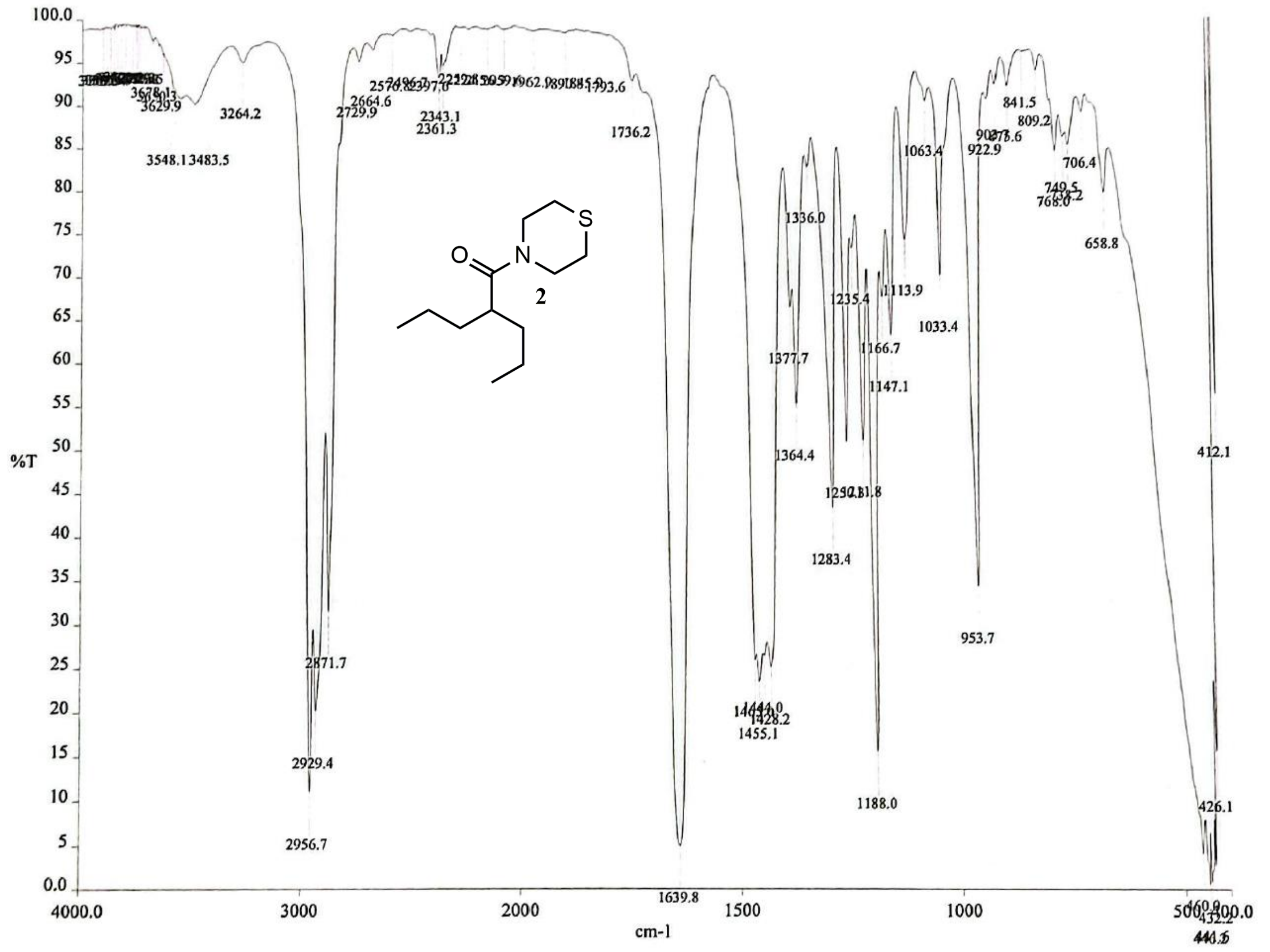

Figure S6. IR spectrum (KBr disc) of compound **2**.

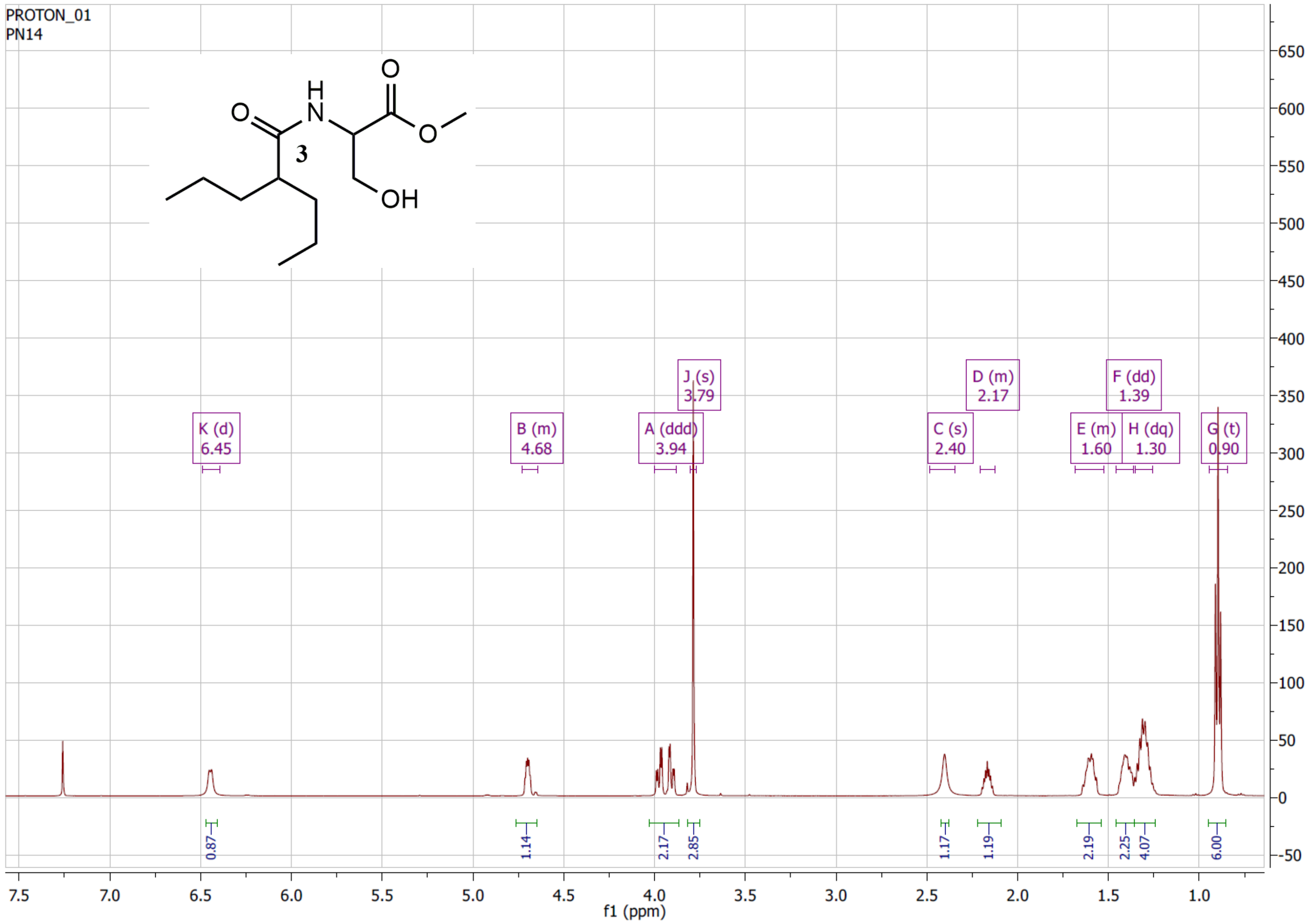

Figure S7. ^{1}H NMR (300 MHz, $CDCl_3$) of compound **3**.

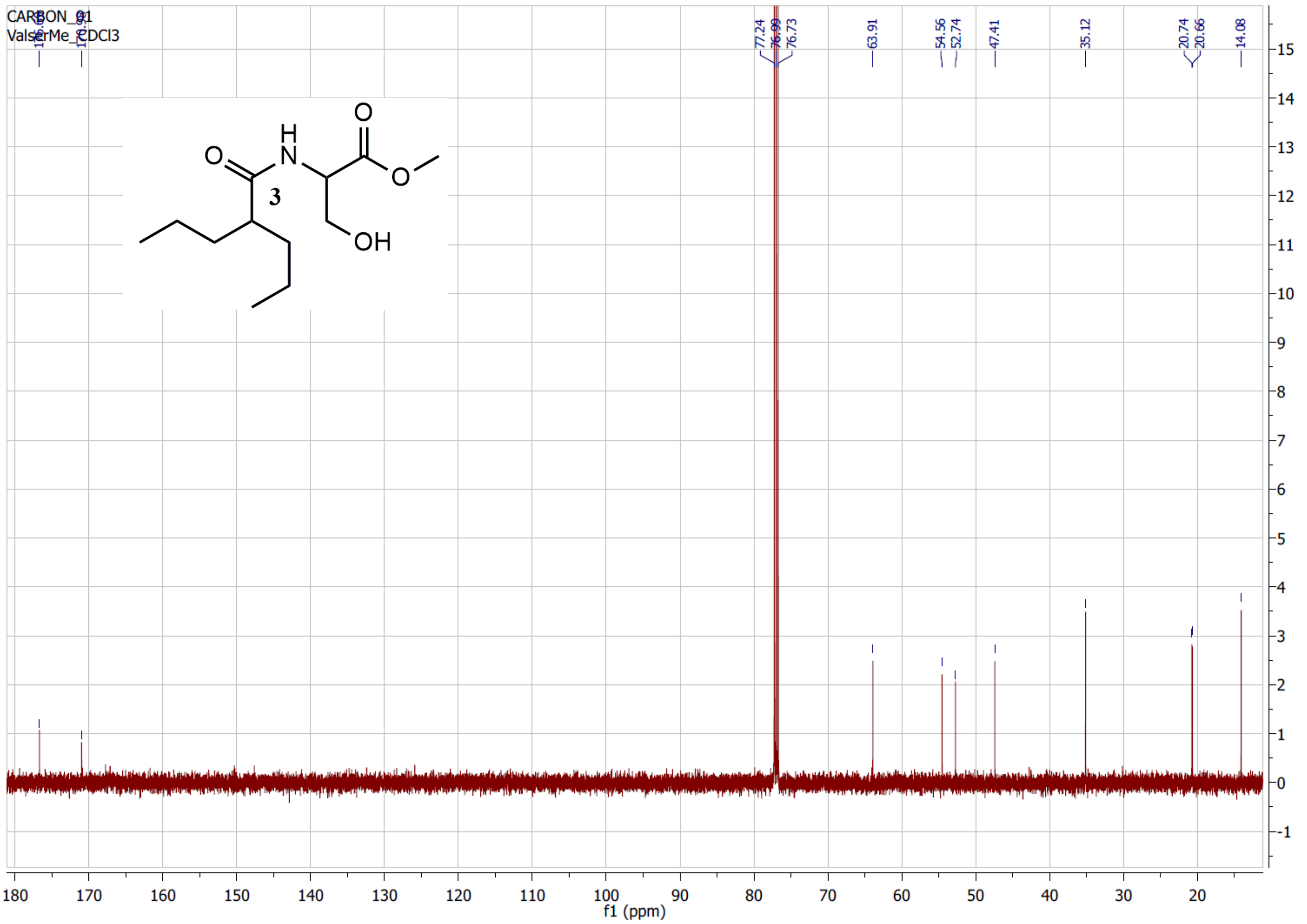

Figure S8. ^{13}C NMR (500 MHz, $CDCl_3$) of compound **3**.

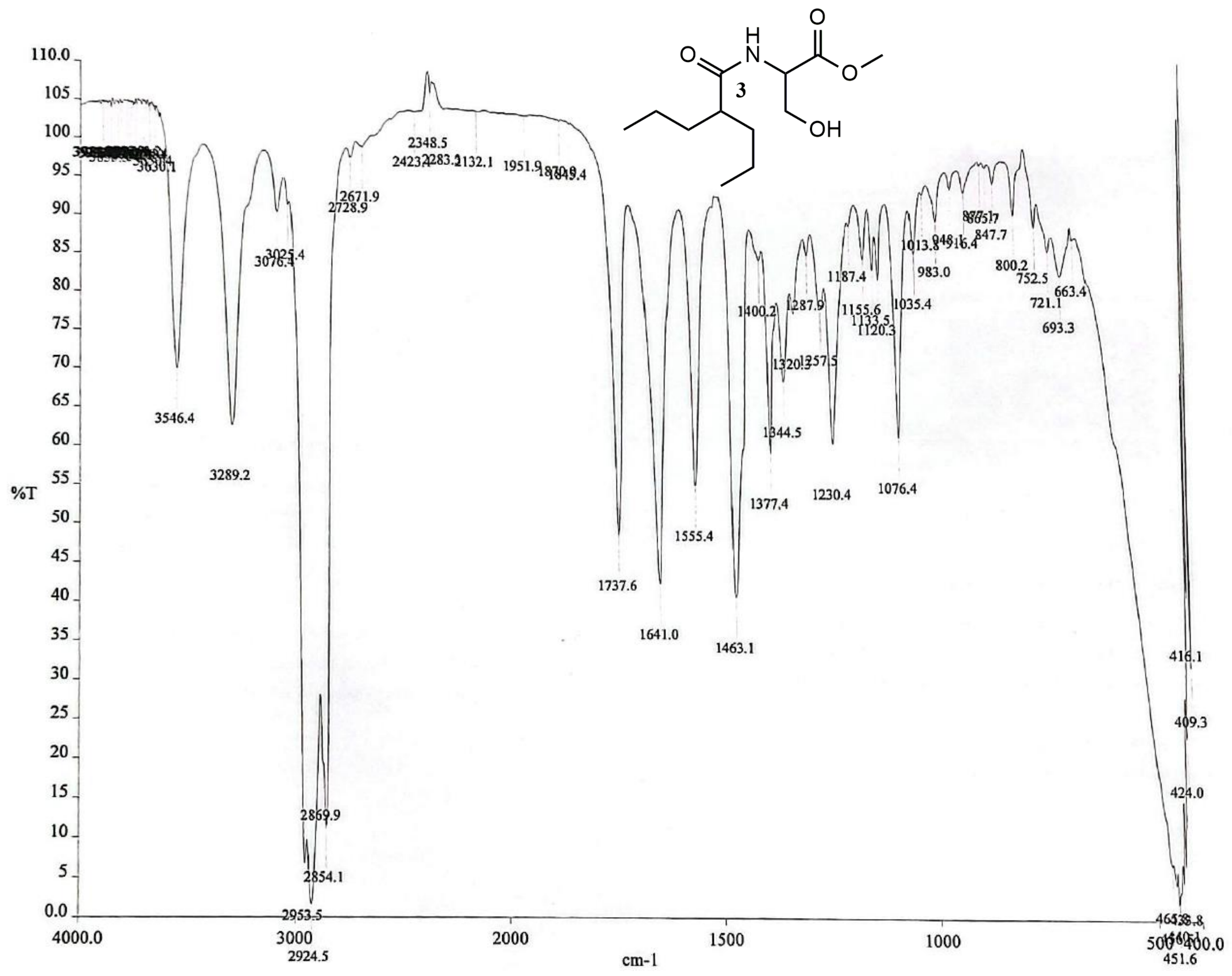

Figure S9. IR spectrum (Nujol) of compound **3**.

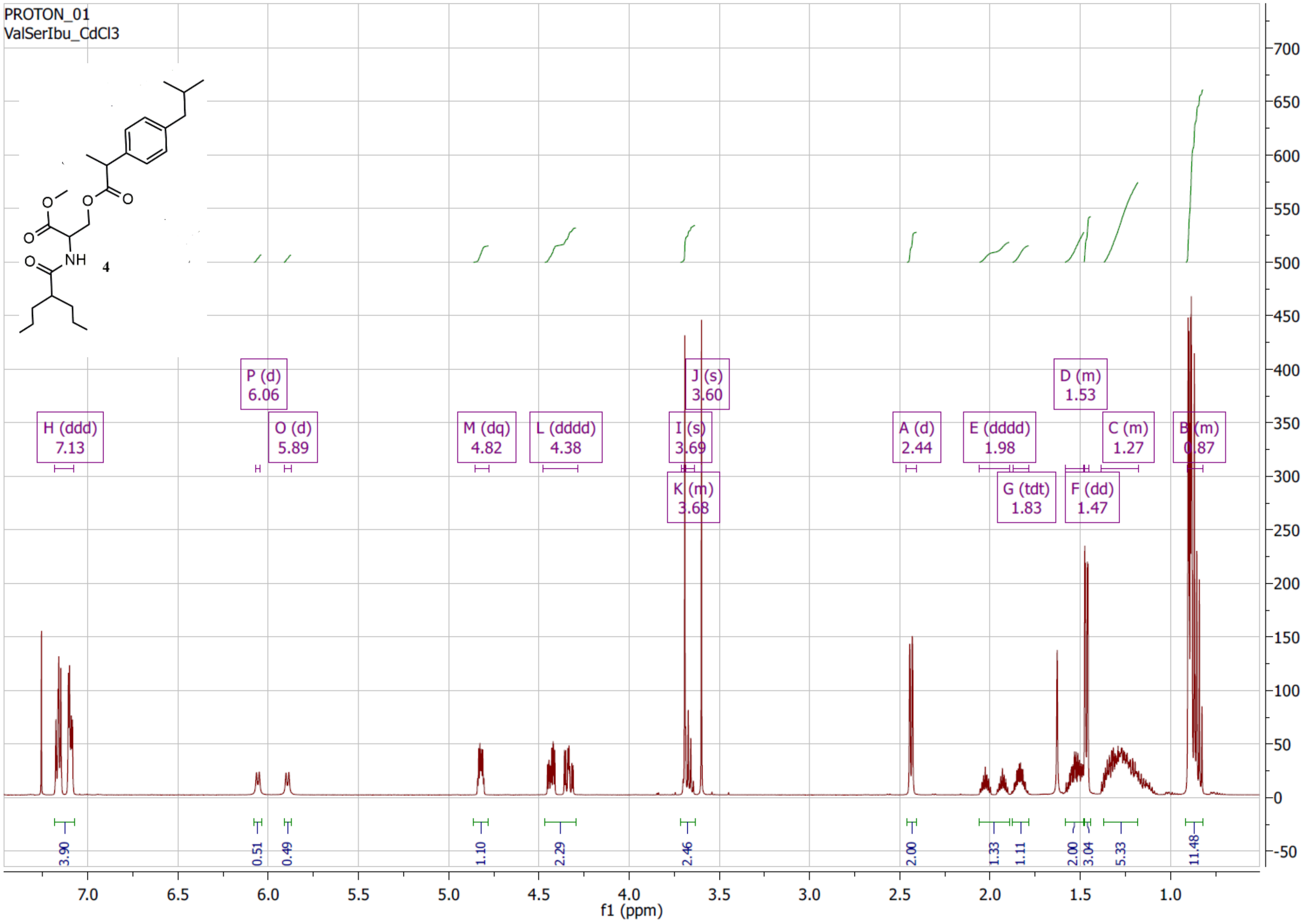

Figure S10. ^{1}H NMR (500 MHz, $CDCl_3$) of compound **4**.

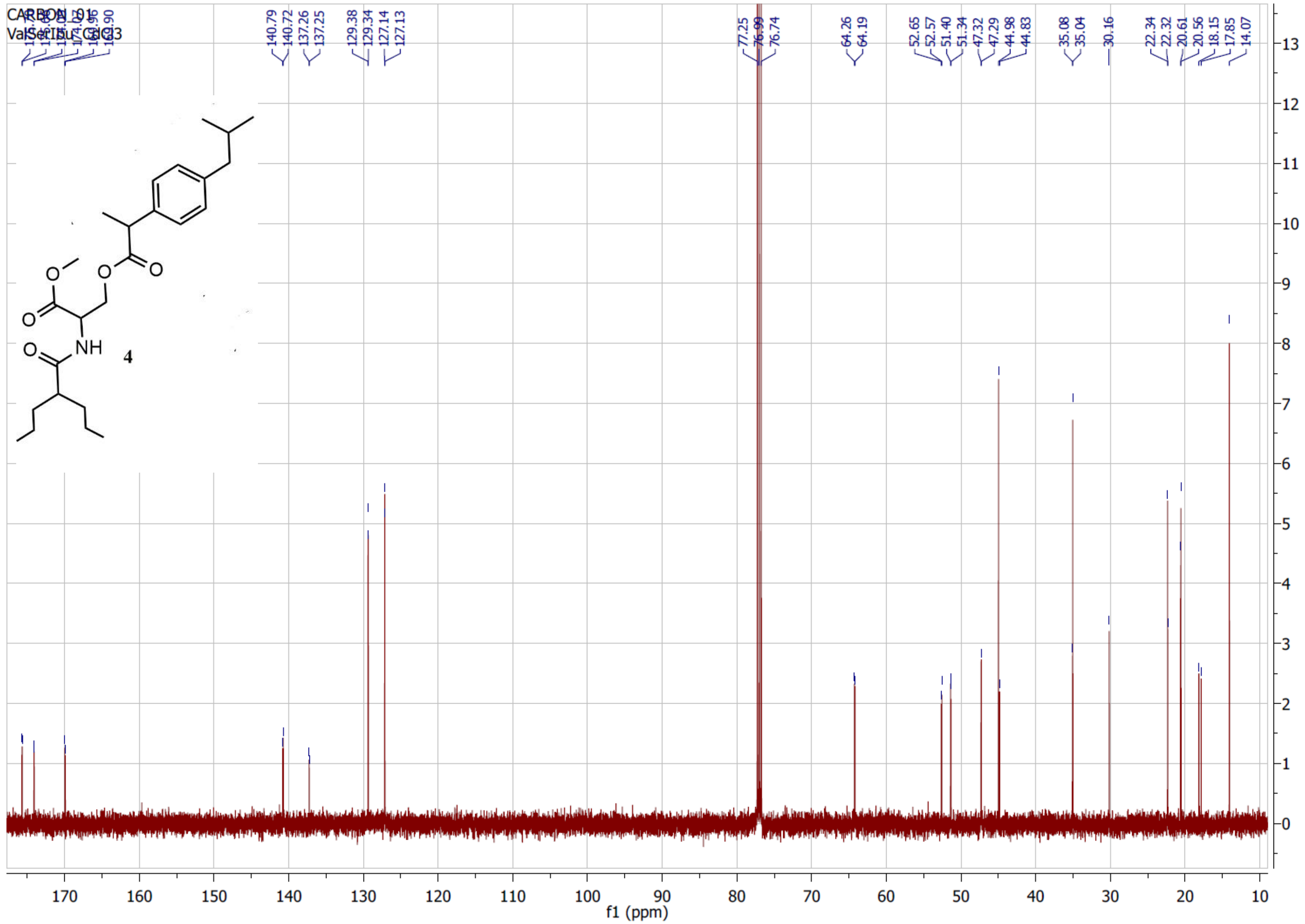

Figure S11. ^{13}C NMR (500 MHz, $CDCl_3$) of compound **4**.

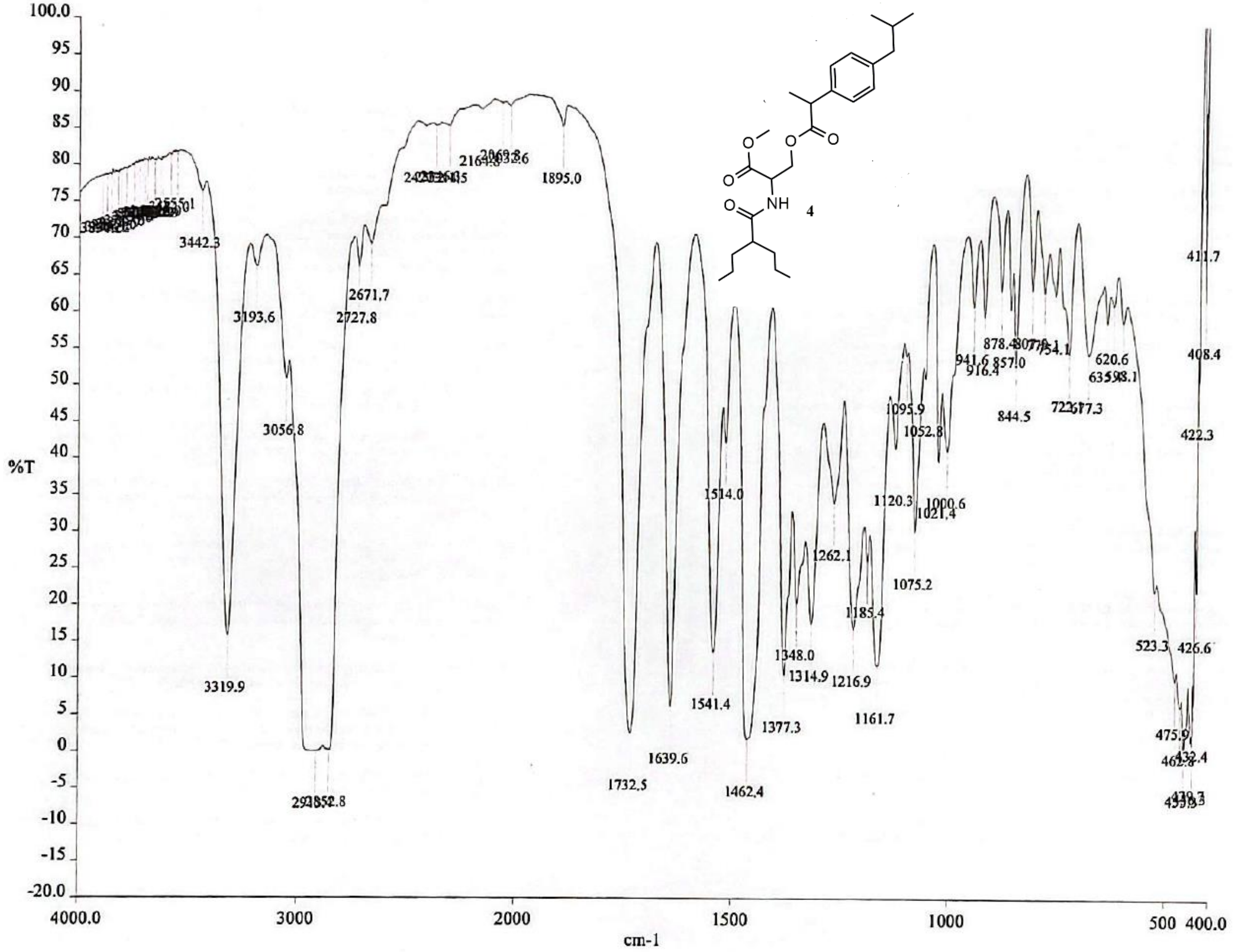

Figure S12. IR spectrum (Nujol) of compound **4**.

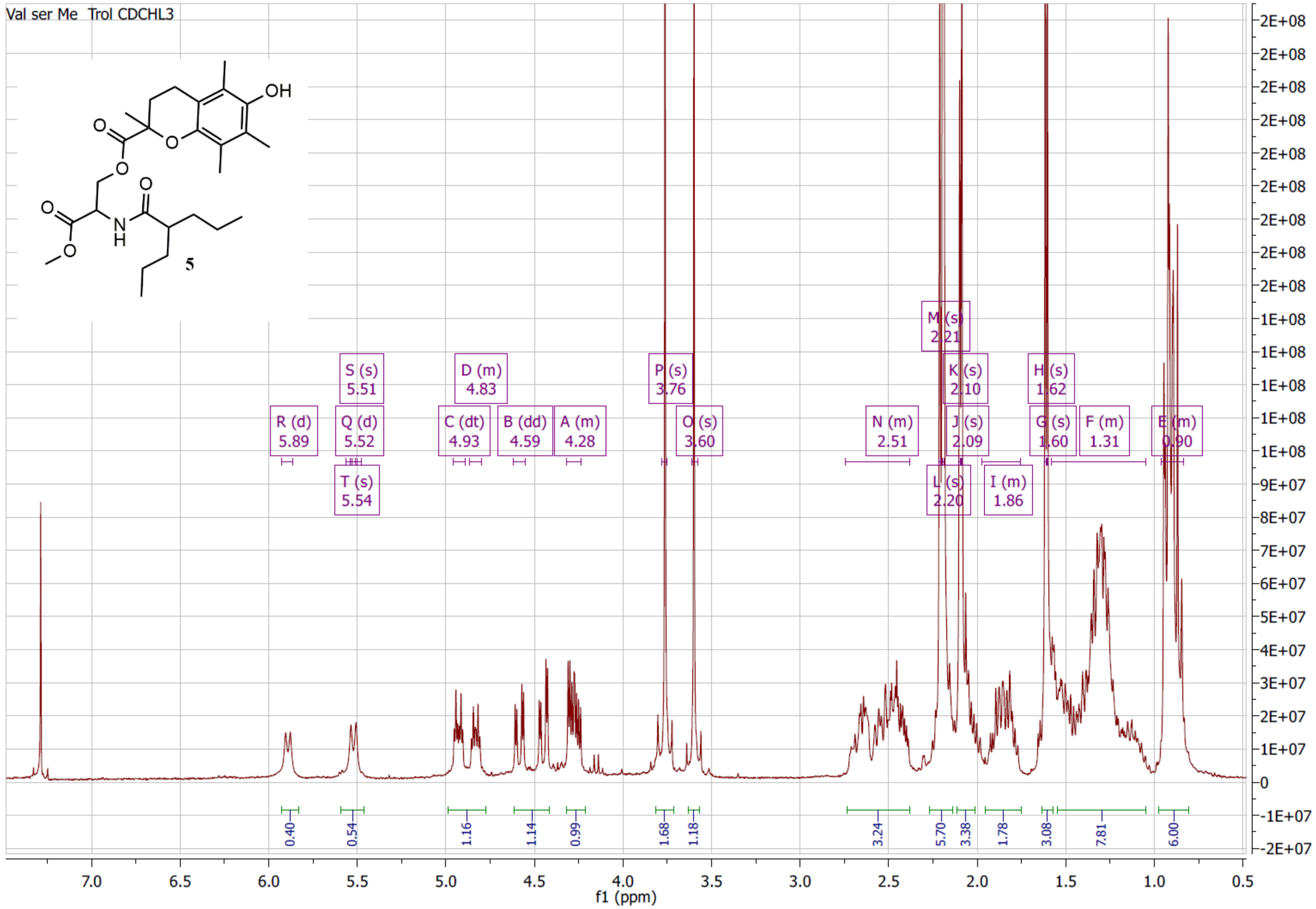

Figure S13. ^{1}H NMR (300 MHz, $CDCl_3$) of compound **5**.

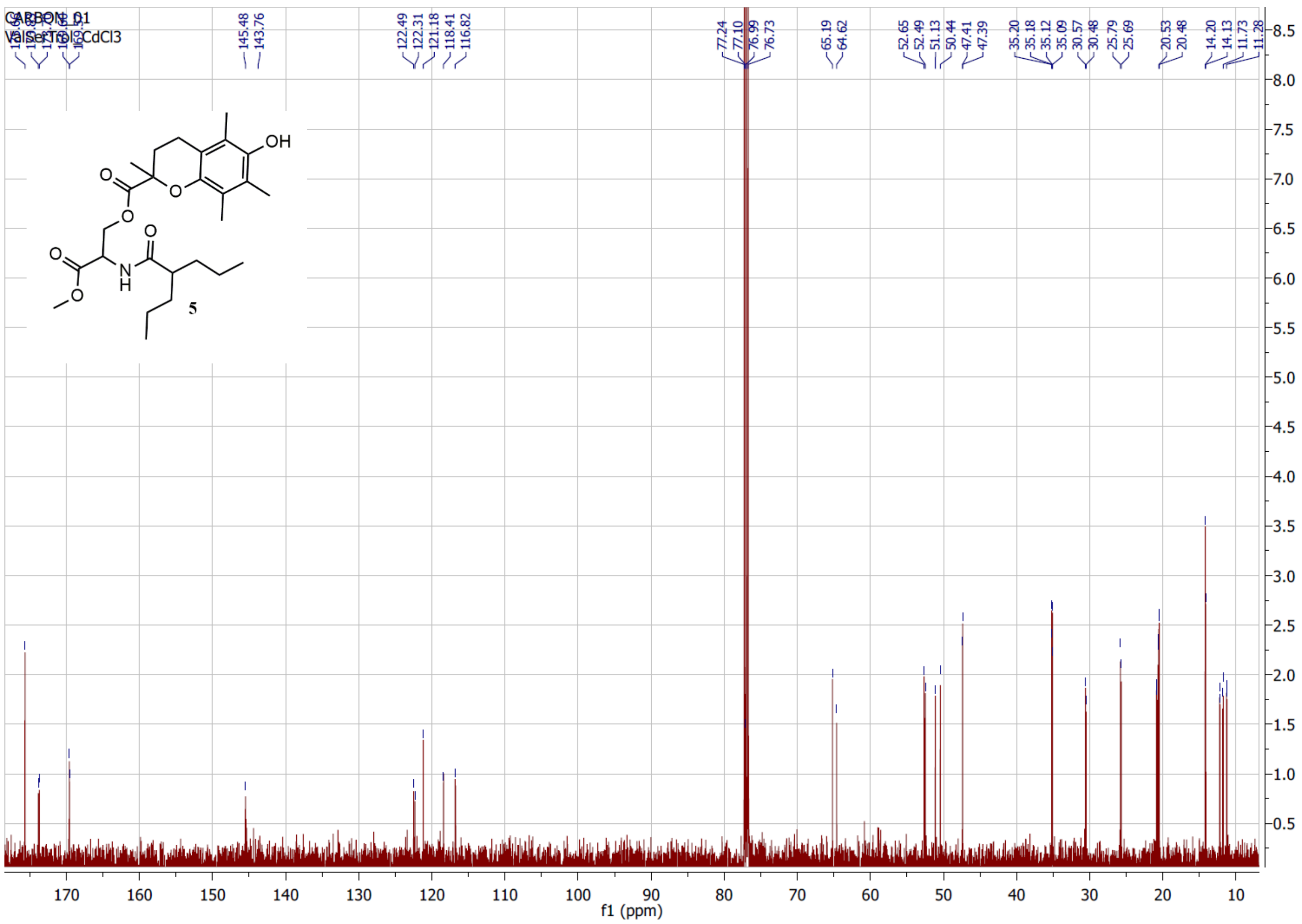

Figure S14. ^{13}C NMR (500 MHz, $CDCl_3$) of compound **5**.

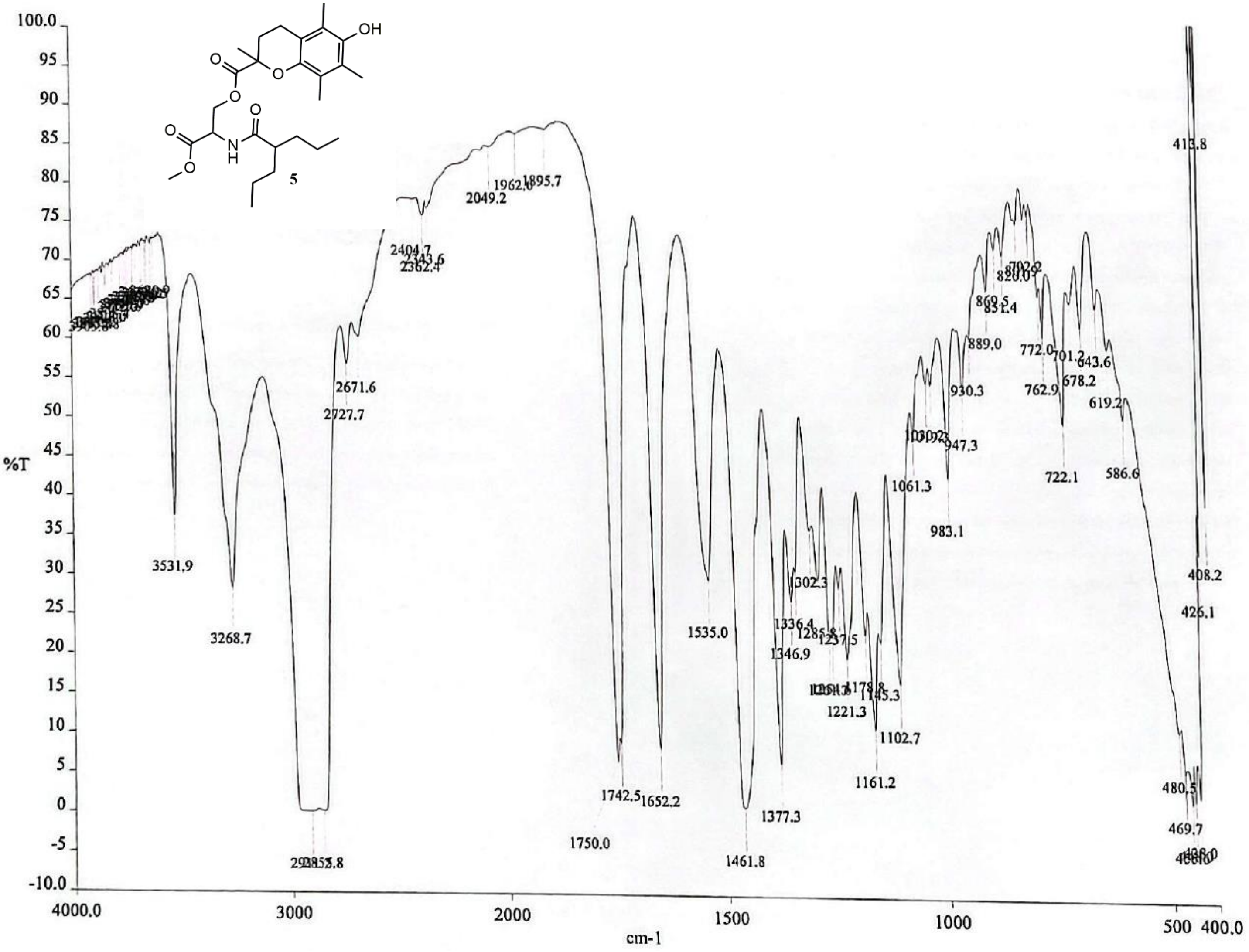

Figure S15. IR spectrum (Nujol) of compound **5**.

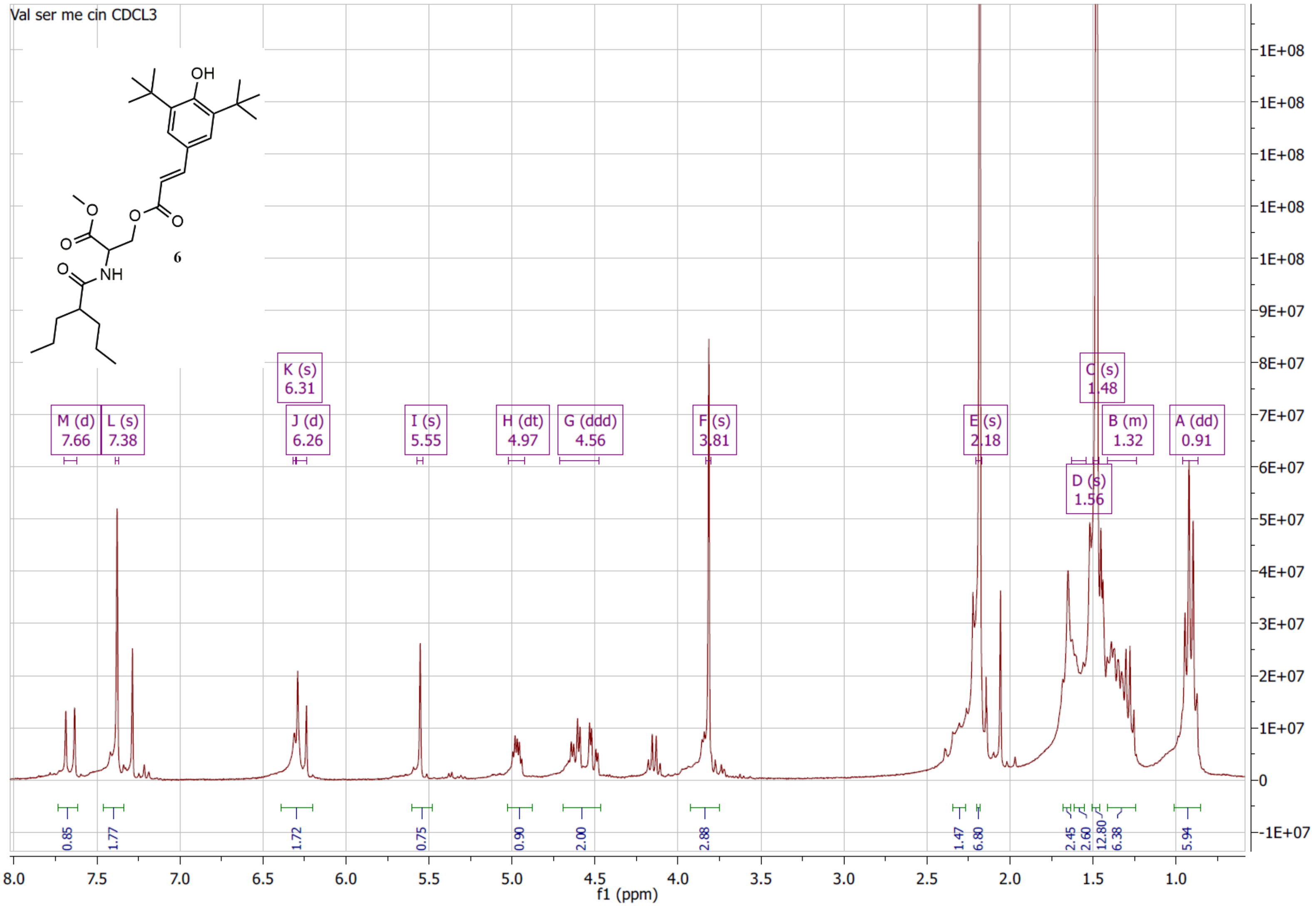

Figure S16. ^{1}H NMR (300 MHz, $CDCl_3$) of compound **6**.

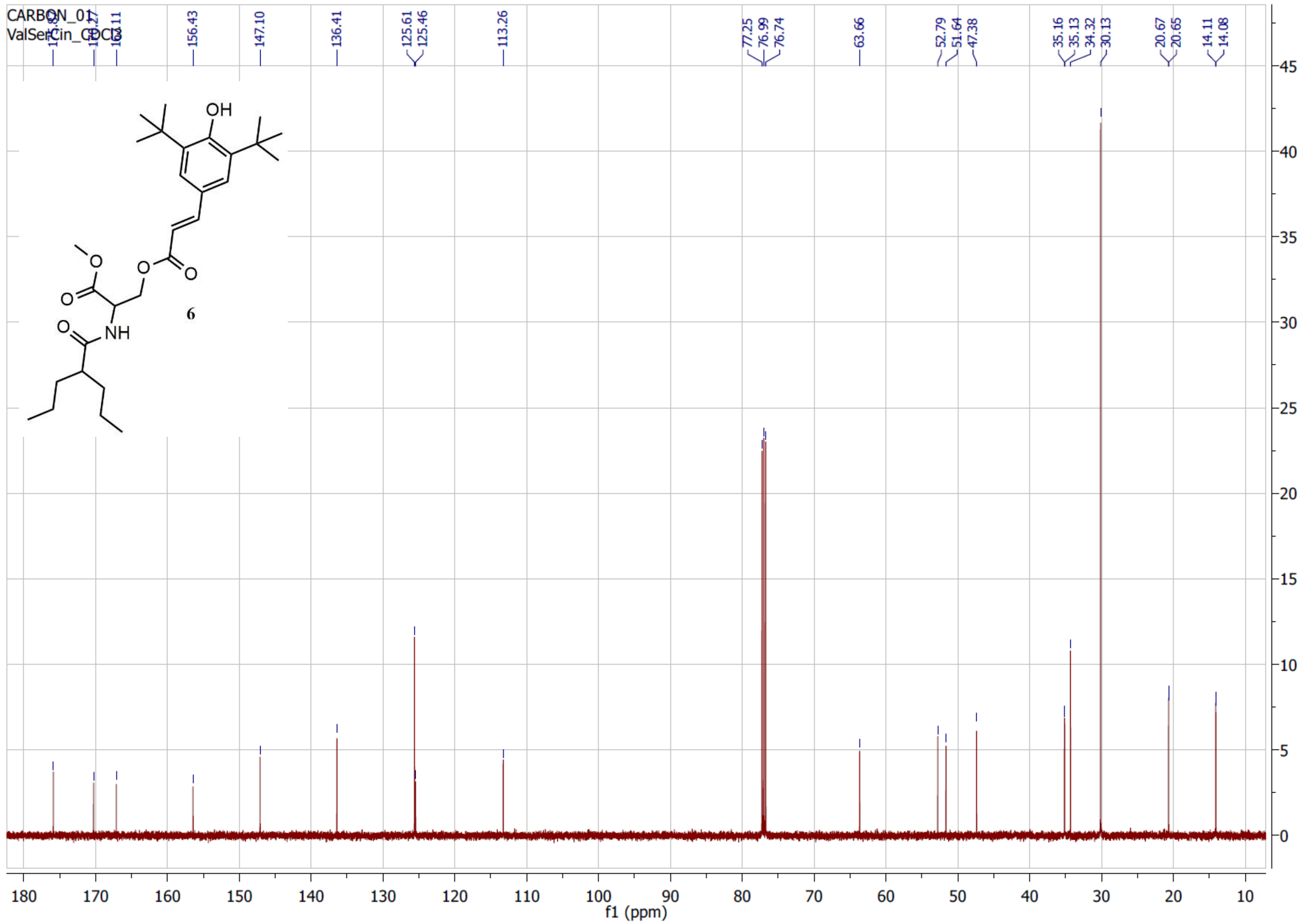

Figure S17. ^{13}C NMR (500 MHz, $CDCl_3$) of compound **6**.

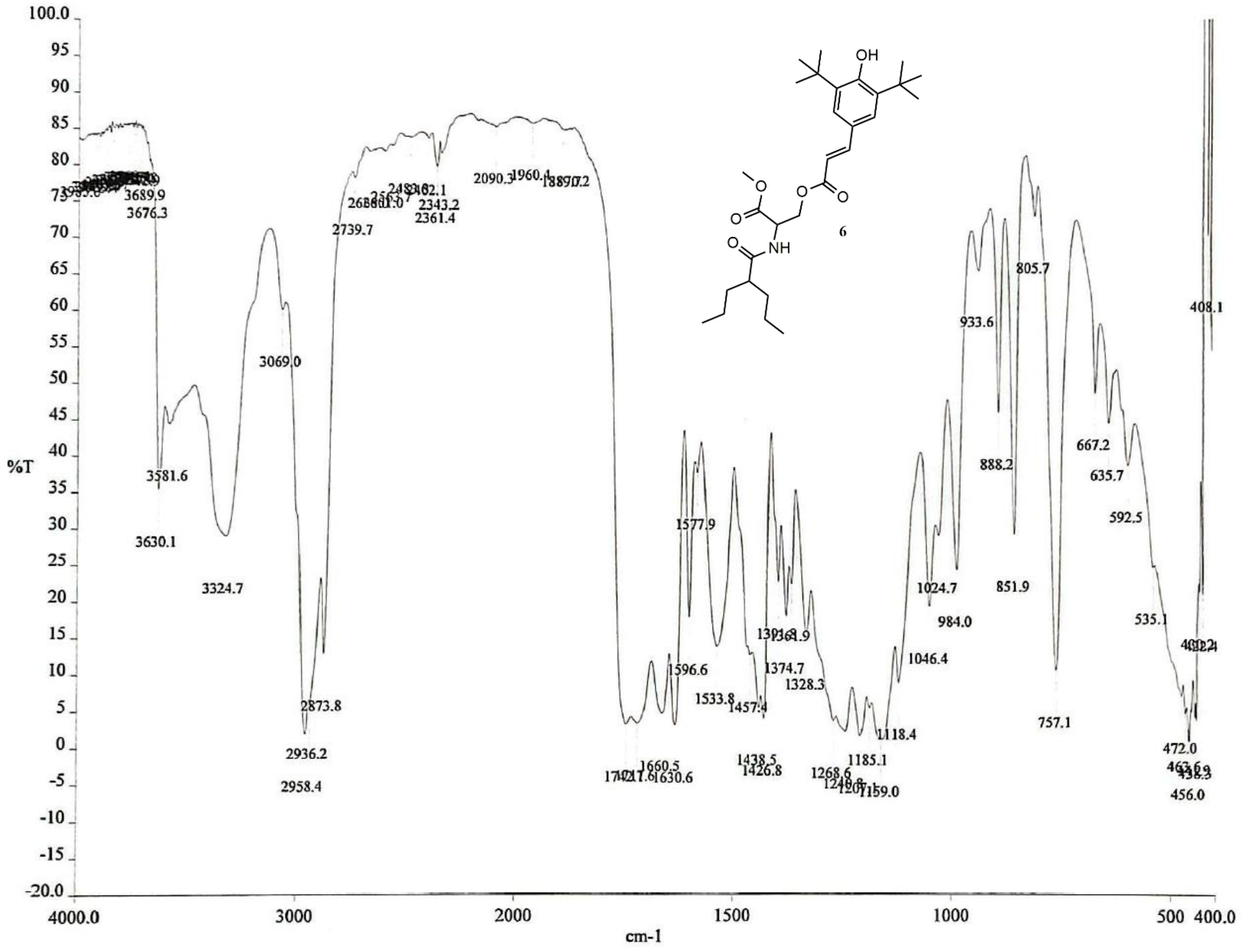

Figure S18. IR spectrum (KBr disc) of compound **6**.
